# Innovative Hybrid-Alignment Annotation Method for Bioinformatics Identification and Functional Verification of a Novel Nitric Oxide Synthase in *Trichomonas vaginalis*

**DOI:** 10.3390/biology11081210

**Published:** 2022-08-12

**Authors:** Hung-Che Lin, Hao-Ai Shui, Kuo-Yang Huang, Wei-Zhi Lin, Hsin-Yi Chang, Hwei-Jen Lee, Ying-Chih Lin, Yuahn-Sieh Huang, Guan-Ru Chen, Ya-Ting Yang, Hsiu-Lin Liu, Yi-Syuan Wu, Chia-Shiang Cheng, Ching-Lung Ko, Yu-Tien Chang, Jih-Chin Lee, Chen-Shien Lin, Chih-Hung Wang, Chi-Ming Chu

**Affiliations:** 1 Graduate Institute of Medical Sciences, National Defense Medical Center, Taipei 114, Taiwan; 2 Department of Otolaryngology-Head and Neck Surgery, Tri-Service General Hospital, National Defense Medical Center, Taipei 114, Taiwan; 3 Graduate Institute of Pathology and Parasitology, National Defense Medical Center, Taipei 114, Taiwan; 4 Graduate Institute of Life Sciences, National Defense Medical Center, Taipei 114, Taiwan; 5 Department of Research and Development, National Defense Medical Center, Taipei 11490, Taiwan; 6 Department of Biochemistry, National Defense Medical Center, Taipei 114, Taiwan; 7 School of Public Health, National Defense Medical Center, Taipei 114, Taiwan; 8 Department of Biology and Anatomy, National Defense Medical Center, Taipei 114, Taiwan; 9 Chinese Medicine Department, Taipei Hospital, Ministry of Health and Welfare, New Taipei City 242, Taiwan; 10 Big Data Research Center, College of Medicine, Fu-Jen Catholic University, New Taipei City 242, Taiwan; 11 Department of Public Health, Kaohsiung Medical University, Kaohsiung 807, Taiwan; 12 Department of Public Health, China Medical University, Taichung 406, Taiwan

**Keywords:** bioinformatic, gene annotation, novel nitric oxide synthase, *Trichomonas vaginalis*, protein structure prediction

## Abstract

**Simple Summary:**

Both the annotation and identification of genes in pathogenic parasites remain challenging. As a survival factor, nitric oxide (NO) has been proven to be synthesized in *Trichomonas vaginalis* (TV). However, nitric oxide synthase (NOS) has not yet been annotated in the TV genome. By aligning whole coding sequences of TV against a thousand sequences of known proteins from other organisms via the Smith–Waterman and Needleman–Wunsch algorithms, we developed a witness-to-suspect strategy to identify incorrectly annotated genes in TV. A novel NOS of TV (TV NOS) with a high witness-to-suspect ratio, which was originally annotated as a hydrogenase in the NCBI database, was successfully identified. We then performed in silico modeling of the protein structure and the molecular docking of all cofactors (NADPH, tetrahydrobiopterin (BH4), heme and flavin adenine dinucleotide (FAD)), cloned the gene, expressed and purified the protein, and ultimately performed mass spectrometry analysis and enzymatic activity assays. We clearly showed that although the predicted structure of TV NOS is not similar to that of NOS proteins of other species, all cofactor-binding motifs can interact with their ligands with high affinities. Most importantly, the purified protein is a functional NOS, as it has a high enzymatic activity for generating NO in vitro. This study provides an innovative approach to identify incorrectly annotated genes.

**Abstract:**

Both the annotation and identification of genes in pathogenic parasites are still challenging. Although, as a survival factor, nitric oxide (NO) has been proven to be synthesized in *Trichomonas vaginalis* (TV), nitric oxide synthase (NOS) has not yet been annotated in the TV genome. We developed a witness-to-suspect strategy to identify incorrectly annotated genes in TV via the Smith–Waterman and Needleman–Wunsch algorithms through in-depth and repeated alignment of whole coding sequences of TV against thousands of sequences of known proteins from other organisms. A novel NOS of TV (TV NOS), which was annotated as hydrogenase in the NCBI database, was successfully identified; this TV NOS had a high witness-to-suspect ratio and contained all the NOS cofactor-binding motifs (NADPH, tetrahydrobiopterin (BH4), heme and flavin adenine dinucleotide (FAD) motifs). To confirm this identification, we performed in silico modeling of the protein structure and cofactor docking, cloned the gene, expressed and purified the protein, performed mass spectrometry analysis, and ultimately performed an assay to measure enzymatic activity. Our data showed that although the predicted structure of the TV NOS protein was not similar to the structure of NOSs of other species, all cofactor-binding motifs could interact with their ligands with high affinities. We clearly showed that the purified protein had high enzymatic activity for generating NO in vitro. This study provides an innovative approach to identify incorrectly annotated genes in TV and highlights a novel NOS that might serve as a virulence factor of TV.

## 1. Introduction

With advances in next-generation sequencing (NGS) and the falling cost of genome sequencing, sequence annotations in gene identification and prediction remain challenging [1]. In addition, few of these predictions have been curated experimentally. The sequence-to-function gap is still a challenge for scientists, despite tremendous increases in sequencing speed [2]. Genome annotation involves two types of processes. One is genome structure annotation, which identifies genes and their intron–exon relationships. The other is genome function annotation, which uses database data, such as gene ontology, for annotations [1]. Through functional annotation to understand the function of sequences in an organism, we can conduct a comprehensive analysis of upstream and downstream information, including new gene discovery, repeated sequence determination, regulatory gene determination, and complete genetic analysis [3]. However, some studies have shown that with the widespread use of systematic automated annotations, the proportion of sequences that are misannotated in public databases is also increasing; thus, future solutions for misannotation are indispensable [4,5].

Many studies indicate that nitric oxide synthase (NOS) is found in a wide range of organisms, including bacteria and animals, and that a few algae and protists have similar NOS-like structures or activity [6,7,8,9,10,11]. Although the evolution of NOS varies among organisms, they still have similar conserved domains [9]. NOS plays an important role in the reduction–oxidation reaction within organisms, which requires NADPH to provide electrons, catalyzes L-arginine and oxygen, and generates L-citrulline and nitric oxide (NO) [9,12,13]. The activated form of NOS is a homodimer structure, including the NOS oxygenase domain (NOSoxy) with a heme protein and the NOS reductase domain (NOSred) with a cofactor flavoprotein. Within mammals, calmodulin (CaM) transfers electrons from the reductase domain to the oxygenase domain [7,12,13,14,15]. In addition, NO is related to a number of important physiological functions in mammals, including vasoconstriction, nerve conduction, and immune response [16]. Studies have shown that the NOS genes and protein products in animals have similar characteristics (e.g., 56.3–60.9% identity in NOS sequences in humans) and the intron position in the NOS gene is also highly conserved, indicating that they may derive the NOS gene of *Metazoa* [16]. A protein in the protist *Physarum polycephalum* acts similarly to mammalian NOS during spore formation. Although its sequence identity to known NOS is less than 39%, it contains a conserved binding domain common to all NOS [13,17]. Among photosynthetic autotrophs, *Ostreococcus tauri* is the earliest algae that has been found to have NOS activity; the protein has a similarity of 44–49% to human NOS and contains both NOSoxy and NOSred domains [8,14]. Bacteria can produce NO via bacterial NOS (bNOS) and different types of bacteria have different purposes for NO. In addition to UV resistance, against oxidative and antibiotic stress, NO is also involved in synthesizing nitro compounds, as well as transcription adjustment and other functions [9,18,19]. Most bNOS structures consist of the NOSoxy domain alone and may obtain electrons from endogenous NAD(P)H reductase, but a few bNOS are multidomain.

*T. vaginalis* (TV) is a microaerophilic unicellular protozoan that parasitizes the human urogenital tract epithelium and its genome was completely sequenced in 2007 [20]. With six chromosomes, the number of genes is predicted to be approximately twice that of a human and it also possesses highly repetitive gene sequences. It is speculated to adapt to changes in the urogenital environment [20]. In 2006, Harris et al. [21] proposed the presence of NOS activity in two microaerophilic parasites, including TV and *Giardia intestinalis*. They found that under aerobic conditions, TV displays NOS activity and turns arginine into citrulline and nitric oxide [6,22]. The two species of anaerobic protists are similar in their evolutionary classification [22]. In addition, Harris et al. [6,21] proposed that the sequence structure may encode two NOS isoforms. In 2007, Morrison et al. [23] reported that *G. intestinalis* uses the arginine dihydrolase (ADH) pathway to metabolize arginine and perform NO biosynthesis. The ADH pathway in TV was noted by Carlton et al. [20] in 2007. Arginine is also an important source of energy. TV is a kind of eukaryote, but it lacks mitochondria and alternatively has a hydrogenosome for fermentative carbohydrate metabolism. Studies in the past found that the hydrogenosome of TV turns arginine into citrulline and ammonia through arginine deiminase (ADI). This step is a part of the energy production process of putrescine biosynthesis through the ADH pathway [24,25]. In addition, TV hydrogenosomes produce more NO and enhance hydrogenosomal membrane potential in an iron-restricted environment to maintain cell survival [26].

The gene annotation of NOS in TV has not been validated in the past 2 decades, although the gene annotation of *G. intestinalis* was completed in 2010 [23,27,28]. Therefore, the blastp (protein–protein BLAST) results were obtained from NCBI using NOS of *G.* *intestinalis*. Unfortunately, the closest comparison with other species was found to be iNOS in house mice, but no sequence similar to that in TV was found. Hence, there is still little information available regarding NOS in TV [21]. Here, we use a hybrid annotation method by combining the Smith–Waterman algorithm and the Needleman–Wunsch algorithm to systematically explore and annotate NOS in TV. In the Smith–Waterman algorithm, the similarity of partial regions is a priority and has higher sensitivity, which is used for the comparison of cross-species proteins. In the Needleman–Wunsch algorithm, the sequences of two pairs are aligned, regardless of their length, and compared from beginning to end, which can be used for comparison of highly similar sequences. By using these methods, our study hopes to identify the novel NOS gene in TV.

## 2. Materials and Methods

### 2.1. Sequence Retrieval, Data Reduction, Witness-vs.-Suspect Search, and Hybrid-Alignment Annotation Method

**Figure 1:**
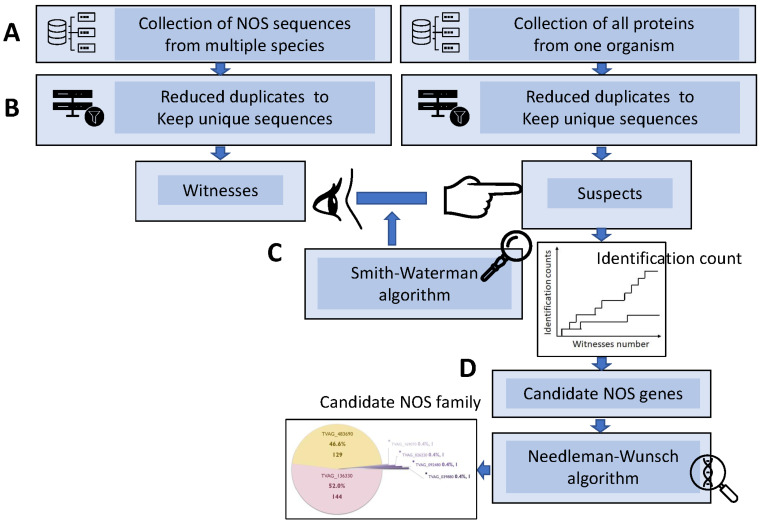
Flowchart illustrating the strategy and procedure of identifying TV NOS genes in this study. (**A**) Protein sequences of non-TV organisms were retrieved from NCBI database to serve as witnesses, while sequences of all TV proteins were retrieved to serve as suspects. (**B**) Duplicates of the retrieved sequences were removed to keep unique sequence for each witness or suspect. (**C**) Smith–Waterman algorithm were performed as the first step of the hybrid alignment method to identify suspects with a score greater than 100 bits by using non-TV NOSs as query sequences. (**D**) Needleman–Wunsch algorithm was performed as the second step of the hybrid alignment method to determine the final candidates with high witness-to-suspect ratios.

Traditionally, the process of gene annotation in an organism is to use gene sequences of that organism as queries to search against all annotated genes of other species in the database. Misannotation sometimes occurs when a query sequence is analogous to more than one family of genes. To avoid the ambiguous situation, we used a reverse approach to focus on identifying only one gene family in one species in which the gene family has never been identified. In this study, a witness-to-suspect strategy was used to dig out the unidentified NOSs in Trichomonas vaginalis. In this way, we can remove interference from the sequences of unrelated genes during gene identification. As shown in the flowchart in Figure 1, our approach can be divided into four steps.

First, we collect sequences of a single-protein family, such as annotated nitric oxide synthase from all species to serve as queries (i.e., witnesses) and collect whole sequences (i.e., suspects) of a single organism such as *Trichomonas vaginalis* as subjects (Figure 1A). In terms of NOS, sequences of non-Trichomonas-vaginalis NOS from NCBI proteins database were defined as witnesses, while all protein sequences of Trichomonas vaginalis from NCBI proteins database were defined as suspects. As a result, in total, 14,107 NOS sequences from all species and 120,958 TV sequences were downloaded and exported as FASTA files from NCBI protein database.

Second, we used MATLAB to remove duplicates to keep unique sequences(Figure 1B). After removing duplicates from using MATLAB, only 50,769 sequences of Trichomonas vaginalis and 3639 sequences of NOS were left. We finally used the 50,769 and 3639 sequence sets as inputs for analysis.

Third, we performed a witness-to-suspect search with Smith–Waterman (SW) algorithm (Figure 1C, the first step of hybrid-alignment annotation method). Using SW algorithm, query sequences of a single family from multiple species serve as witnesses to identify the suspects belonging to the same gene family from all sequences of a subject single organism [29,30]. MATLAB R2014a software was used to perform the Smith–Waterman algorithm (local alignment) between all TV sequences and all the NOS sequences from other species downloaded from the NCBI protein database. The output results after SW analysis were used as an input into SPSS 22.0 for score sorting. Since each witness sequence was compared and aligned with all suspect sequences of TV, one with highest score can be regarded as one-time identification of a candidate NOS. After sorting from high to low scores, sequences with a score greater than 100 bits, a threshold higher than the requirement of NCBI database (40–50 bits), were regarded as a successful identification. The ratio of identification of candidate NOS can be estimated [30]. The hit numbers of identification for each suspect were summarized as a percentage of all identification. All identified suspects were collected as candidate genes for following global alignments. 

Fourth, Needleman–Wunsch algorithm was used to perform global alignment between the candidates to filter out low-confidence ones and keep the final top candidates [30] (Figure 1D, the second step in the hybrid-alignment annotation method). After years of research in our laboratory, the rule of thumb of our annotation method has proven that a suspect with final witness-to-suspect ratio greater than 30 is a high-confidence suspect with low false-positive identification. A pie chart was constructed to show the identification percentages and a phylogenetic tree was used to find evolutionary relationships between different species by MATLAB. We took the top 32 high-scoring NOS non-TV sequences and two major TV NOS sequences for phylogenetic analysis. The output of MATLAB would show the gene name TVAG_136330.

### 2.2. Structure Prediction and Models of TV

To simulate whole proteins, we used the alphafold2 advanced (https://colab.research.google.com/github/sokrypton/ColabFold/blob/main/beta/AlphaFold2_advanced.ipynb) (accessed on 13 September 2021) and the RoseTTAFold (https://robetta.bakerlab.org/submit.php) for the prediction of the structure (accessed on 7 December 2021). TV NOS PDB files were created.

### 2.3. Predicting Domains Using a Bioinformatics Tool and Manually

The TV NOS sequences were annotated on the NCBI database as iron-only hydrogenase large subunit, C-terminal domain containing protein. This means that this protein sequence function is considered to be a hydrogenase by NCBI. The results of the two domain sequences in NCBI and InterPro are shown in the Supplementary Results [31].

The TV NOS FASTA sequences predicted with the NOS function were submitted to the MPI Bioinformatics Toolkit (https://toolkit.tuebingen.mpg.de) (accessed on 15 November 2020) by the MUSCLE alignment tool and TV NOS was aligned with 4 *A. flavus* NOS [32]. Manual alignment with three human NOS domain sequences according to an article published by Foresi was also arranged [8].

### 2.4. Predicting Calmodulin Binding Sites Using the Calmodulin Target Database and HDOCK Server

The amino acid sequences of TV NOS were submitted to the HDOCK server (http://hdock.phys.hust.edu.cn/) (accessed on 4 December 2021) to find probable calmodulin binding sites. This tool allots it to predict the highest affinity for calmodulin binding. Additionally, by using PDB created by alphafold2, protein–protein interactions were tested by the HDOCK server.

### 2.5. Predicting Cofactor Binding Sites Using AutoDock FR Software

The PDB of TV NOS was submitted to AutoDock FR software (https://ccsb.scripps.edu/adfr/) (accessed on 17 November 2021) to find probable cofactor binding sites (including heme, BH_4_, FMN, FAD, and NADP). The manual alignment of the binding sites will serve as a reference for the results of biding sites predicted by AutoDockFR.

### 2.6. Cloning of TV Nitric Oxide Synthase (TVAG_136330) Using a PCR-Based Accurate Synthesis Method

DNA synthesis and gene cloning were conducted by a custom service provided by Elabscience. The nucleotide coding region of TVAG_136330 was selected for full-length TV NOS synthesis based on the published method called PAS (PCR-based Accurate Synthesis). Briefly, multiple overlapping oligonucleotides (60 mer each) which cover the whole length of coding region of TVAG_136330 were designed and chemically synthesized. A nested PCR-based ligation and amplification of the overlapping fragments were performed to gradually elongate the length of the coding regions. Subsequently, a forward primer with BamHI site and a reverse primer with HindIII site were used to produce the full-length TV NOS with start codon with restriction site sequence BamHI and stop codon with restriction site sequence HindIII. Finally, after digestion with HindIII and BamHI restriction enzymes, the PCR product was ligated to vector pFastBac1 (Invitrogen, Waltham, MA, USA) between BamHI-HindIII sites and transformed to Escherichia coli TOP10 strain. The orientation and sequence of inserted TV NOS was verified by Sanger DNA sequencing. 

### 2.7. Subcloning of Gene and Expression of Recombinant TV NOS Proteins

Subcloning, protein expression, and purification were also conducted by a custom service provided by Elabscience. Bac-to-Bac baculovirus expression system (Cat. No. 10359-016, Invitrogen, Waltham, MA, USA) was used to perform subcloning of gene and expression and purification of histidine-tagged recombinant TV NOS proteins. First, the pFastBac1 construction obtained previously was used to transform *E. coli* strain DH10Bac to yield recombinant Bacmids through DNA recombination in the *E. coli* cells. Second, purified recombinant Bacmids were then prepared by a standard method and transfected Spodoptera frugiperda (Sf) insect cells with the virus stock. For expression of recombinant TV NOS, baculovirus was amplified for three generations and infected to Sf9 cells cultured in 200 mL of 1 × 10^6^ cells/mL SF cells. After three days post infection of SF cells, the cells were lyzed and the lysate was centrifugated to remove insoluble debris. Recombinant TV NOS proteins were then purified by a standard affinity absorption to Nichel resins in a column, washed with phosphate-buffered saline, and eluted with imidazole buffer. Finally, the eluted proteins were verified by SDS-PAGE stained with Coomassie blue and Western blotting stained with anti His-Tag antibodies.

### 2.8. Verification of Recombinant TV NOS Proteins by Mass Spectrometry

In addition to verifying the purified TV NOS using SDS-PAGE and Western blot analysis, we further verify the sequence of the purified TV NOS proteins by mass spectrometry. Briefly, fifty micrograms TV NOS protein was dissolved in 6M urea, reduced with 5 mM di-thiothreitol for 45 min at 29 °C, alkylated with 10 mM iodoacetamide in the dark for 45 min at 29 °C, and digested with 1 μg trypsin (MS-grade, Promega, Madison, WI) at 29 °C for 16 h. The reaction was terminated by adding 10% trifluoroacetic acid (TFA) to a final concentration of 0.5% TFA and tryptic peptide solution was desalted with C18 ZipTip prior to LC-MS/MS analysis. The eluted peptides were dissolved in 0.1% formic acid, separated using an Ultimate system 3000 nanoLC system (Thermo Fisher Scientific, Bremen, Germany) equipped with a 75 μm ID, 25 cm length C18 Acclaim PepMap NanoLC column (Thermo Fisher Scientific, San Jose, CA, USA) packed with 2 μm particles with a pore of 100 Å. Mobile phase A was 0.1% formic acid in water and mobile phase B was composed of 100% acetonitrile with 0.1% formic acid. The peptides were eluted at a flow rate of 300 ng/mL with a gradient of 2% to 40% for 90 min. Mass spectra were acquired on a Thermo Scientific™ Orbitrap Fusion™ Lumos™ Tribrid™ Mass Spectrometer (Thermo Fisher Scientific, San Jose, CA, USA). Mass spectrometry analysis was performed in a data-dependent mode with Full-MS (externally calibrated to a mass accuracy of <5 ppm and a resolution of 120,000 at *m*/*z* 200) followed by MS/MS analysis of the most intense ions in 3 s. High-energy collision-activated dissociation (HCD)-MS/MS (resolution of 15,000) was used to fragment multiple charged ions (Charge state 2–7) within a 1.4 Da isolation window at a normalized collision energy of 32. AGC target at 5e5 and 5e4 was set for MS and MS/MS analysis, respectively, with previously selected ions dynamically excluded for 180 s. Max injection time was set as 50 ms. 

Peptides were identified with version v2.0.3.1 against TrEMBL Database (Trichomonas vaginalis version 2022_03). An output MS2 mass spectrometry representative for FMN binding site was obtained and depicted from MaxQuant software (https://www.maxquant.org/) (accessed on 20 March 2022) The sequence coverage of MS was also obtained and depicted from MaxQuant software.

### 2.9. NOS Activity Assay

NOS activity of the cell lysate and purified proteins was measured by a modified Griess reaction using a fluorometric kit (Nitric Oxide Synthase Assay Kit, Fluorometric, Biovision, K206, Milpitas, CA, USA). Protein concentrations of cell lysate and purified proteins were quantified by using Bradford method with bovine serum albumin as the standards. Finally, specific activity of NOS was calculated by normalized against the protein concentrations and converted to activity units according to the manufacturer’s protocol, in which one unit of NOS activity is defined as the amount of enzyme required to yield 1.0 umol of nitric oxide/min at 37 °C.

## 3. Results

### 3.1. Hybrid Annotation Method by Combining the Smith–Waterman Algorithm and the Needleman–Wunsch Algorithm

**Figure 2:**
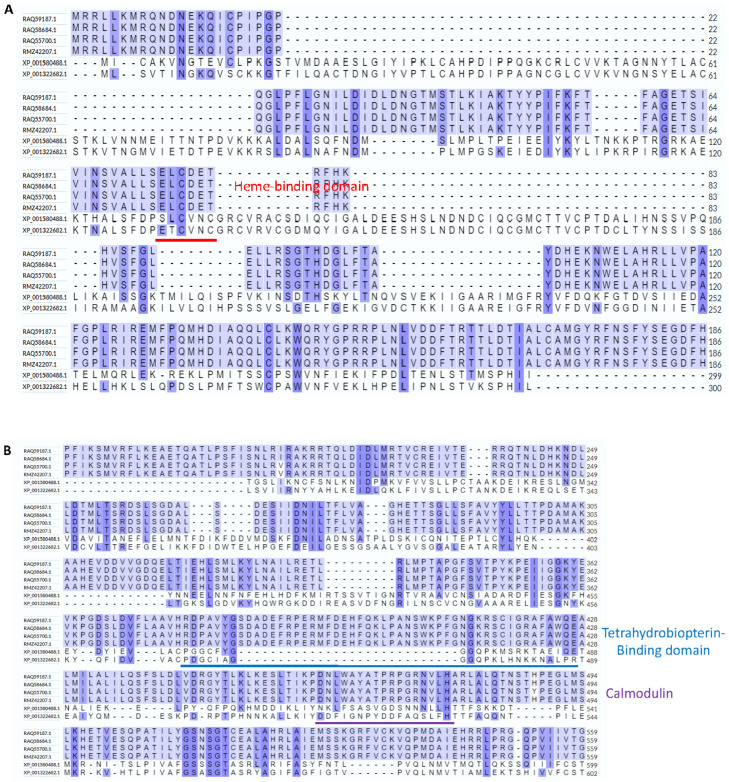

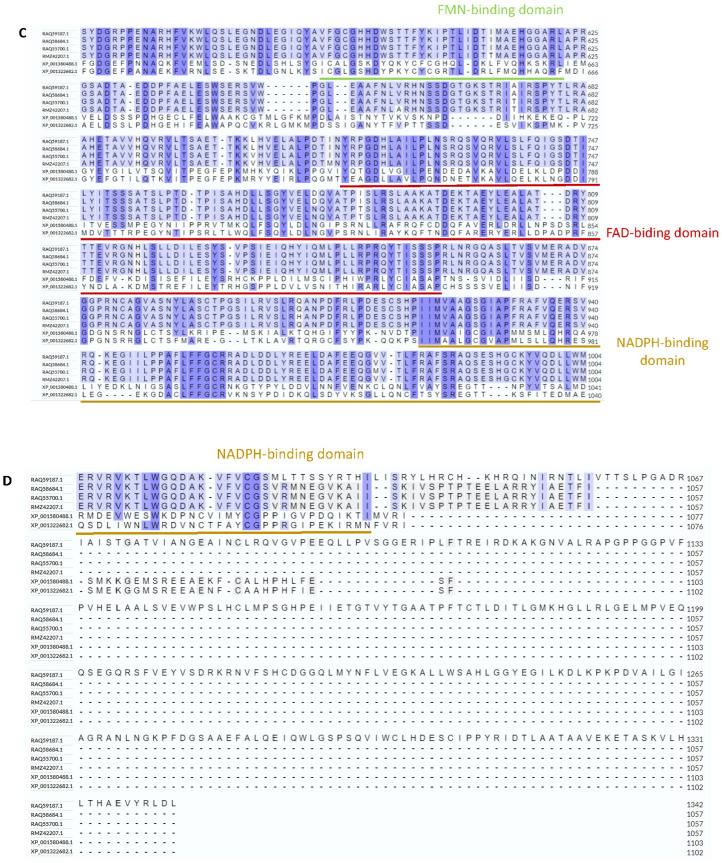
Sequence alignment between two TV NOS and four NOS from *A. flavus*. The alignment was obtained using UniPort alignment tools followed by a manual modification. The conserved NOS domains and motifs, including Heme, BH4, CaM, FMN, FAD, and NADPH binding domains, are marked with blue boxes. (**A**) Heme binding domain prediction binding site (**B**) BH4 domain and CaM prediction biding sites (**C**) FAD and NADPH prediction binding sites (**D**) NADPH prediction binding site.

**Figure 3:**
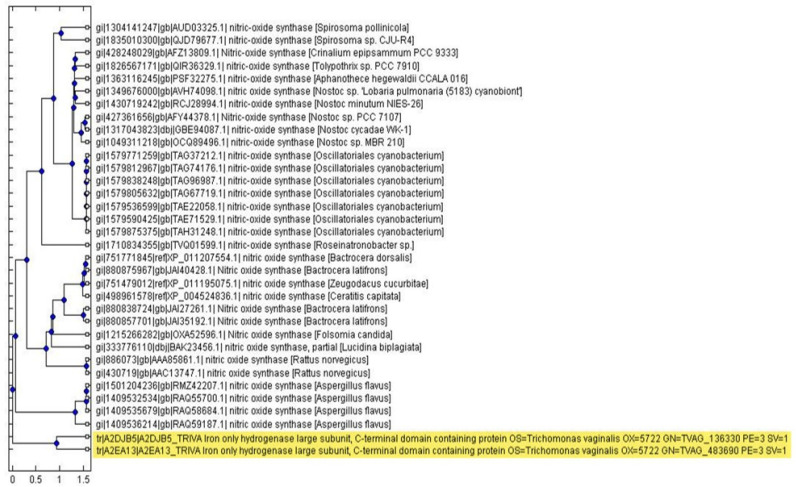
Phylogenetic analysis of TV NOS sequences and non-TV NOS. The phylogenetic evolutional relationship was calculated and depicted by Matlab. Two TV NOS were clustered together, marked by yellow. Note that the closest neighbors are four NOS sequences from *A. flavus*.

By using the Smith–Waterman algorithm and the Needleman–Wunsch algorithm, the results showed a total of 273 identifications of non-TV NOS for searching candidate NOS proteins in TV and the main sequences of TV NOS were recognized. A flowchart of the thought process for this Hybrid Annotation Method of the bioinformatic part is shown in Figure 1. Manual alignment of domain sequences took place according to an article published by Foresi [8]. TV NOS and four Aspergillus flavus NOS protein sequences were submitted to the UniProt website for sequence alignment. Manual alignment of the domain (Heme, BH4, Calmodulin, FAD, FMN, NADPH sites) is shown in Figure 2. The phylogenetic analyses were performed with MATLAB 2014a first, including all top 32 high-scoring NOS protein sequences from other species and TV NOS protein sequences. The results showed that the closest species was four Aspergillus flavus NOS according to phylogenetic analysis (Figure 3). A total of 273 identifications of non-TV NOS stand for searching candidate NOS proteins in TV (Figure S1a). The entire pie chart is evenly distributed, which means that the TV NOS protein sequence is not recognized as candidates with probable NOS function by a single species. In other words, 273 identifications of the non-TV NOS sequences contributed averagely to the identification of the TV NOS protein. In total, six sequences of the TV sequences were recognized as candidates with probable NOS function (Figure S1b). The major genes named TV NOS were recognized. TV NOS revealed the gene names of TVAG_136330.

### 3.2. Protein Structure Prediction and Simulations of TV NOS and by RoseTTAFold and AlpaFold2

**Figure 4:**
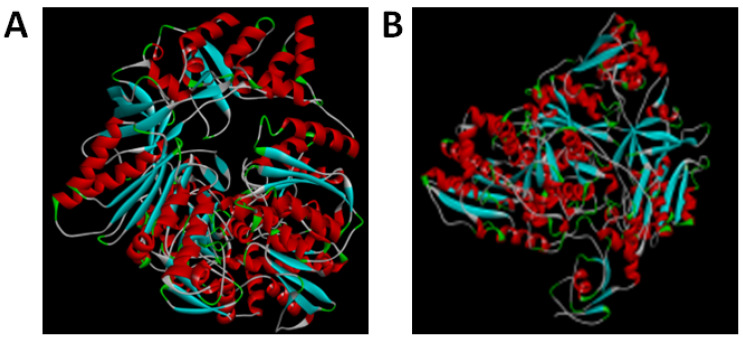
Prediction of three-dimensional conformation of TV NOS by using artificial intelligence programs. Note that the simulation by three-dimensional models predicted by (**A**) Alphafold2 and (**B**) RoseTTAFold was very similar.

With the advancement in artificial intelligence, alphafold and RoseTTAFold can help us to predict the protein structure and perform in silico molecular docking with vital cofactors of NOS (NADPH, BH4, heme, FAD) to reduce unnecessary experimental steps, time, and costs (Figure 4). The RoseTTAFold models were calculated at the server. (https://robetta.bakerlab.org/submit.php) (accessed on 7 December 2021) The confidence scores are like receiver characteristic curves area under curve (ROC AUC) and are used to assess the predicted results. The confidence score 1 revealed good and 0 revealed bad. A value > 0.7 is considered to reflect a reasonable prediction model; a value > 0.8 is considered to reflect a strong prediction model [33]. The result of the TV NOS confidence score by RoseTTAFold was 0.82, which showed that the prediction is a strong candidate for answering queries when the confidence score is greater than 0.7. Protein structure predicted by the Alphafold2 advanced server. (https://colab.research.google.com/github/sokrypton/ColabFold/blob/main/beta/AlphaFold2_advanced.ipynb) (accessed on 13 September 2021) (Figure 4). In 2013, Valerio et al. proposed the Local Distance Difference Test (LDDT) for comparison of protein structures and models by using a local superposition-free score [34]. The pLDDT revealed a predicted local-distance difference test. A higher confidence value represents better results. The pLDDT of Alphafold2 is between 0 and 100. The pLDDT of TV NOS showed 84.05 with confident prediction (confident prediction ranged from 70 to 90 pLDDT). The TM score represents the template modeling score. It reflects the similarity of two proteins. If the protein pairs with a TM-score > 0.5, it reveals that they are mostly of the same fold [35]. The Template Modeling (TM) score of TV NOS was 0.6576, which indicates that the protein has the same fold (TM score > 0.5) (Table 1).

### 3.3. Molecular Docking of TV NOS with Cofactors and NOS Inhibitor (L-NMMA)

**Figure 5:**
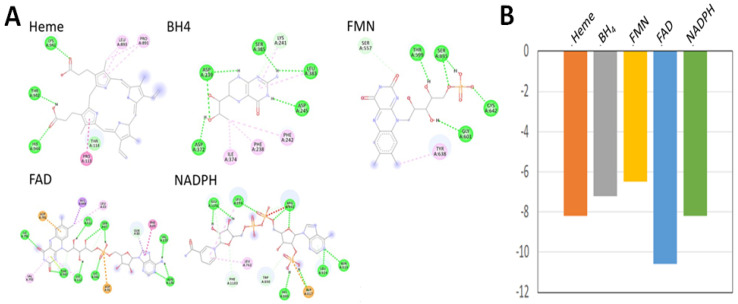
Protein–cofactor interaction sites (**A**) and binding energies (**B**) calculated by autodock FR. The structure of TV NOS used is from prediction of Alphafold2, while the structures of cofactors (Heme, BH4, FMN, FAD, and NADPH) were downloaded from PubChem database. Predicted binding energies for Heme, BH4, FMN, FAD, and NADPH were −7.8, −7.2, −6.5, −10.6, and −8.2 kcal/mol, respectively.

Our docking analysis revealed that all the cofactors (Heme, BH_4_, FMN, FAD, NADPH) exhibited binding energy (TV NOS: heme revealed −7.8 kcal/mol; BH4 revealed −7.2 kcal/mol; FMN revealed −6.5 kcal/mol; FAD −10.6 kcal/mol; NADP −8.2 kcal/mol). NAPDH plays a vital role in NOS activity. [36] Two-dimensional (2D) representation of TV NOS in complex with Heme, BH_4_, FMN, FAD, and NADPH show interactions with conventional H bonds, with interactions further stabilized by different amino acids around the TV NOS backbone. Additionally, docking analysis revealed that the NOS inhibitor (L-NMMA) exhibited binding energy (TV NOS docking with L-NMMA revealed −5.6 kcal/mol). These results suggest that cofactors of NOS can bind to TV NOS protein. In addition, NOS inhibitors can also show good binding affinity to TV NOS protein (Figure 5 and Supplementary Materials, Figures S3–S8).

### 3.4. Prediction of the Protein–Protein Interaction of TV NOS with Calmodulin Protein

Calmodulin plays a vital key role in the physiological processes of NOS. Calmodulin acts as a switch and allows electron transfer from the FMN subdomain to heme. Our in silico protein–protein interaction of TV NOS with calmodulin protein revealed binding sites from amino acids 513 to 527 compatible with our manual alignment. The docking energy scores revealed −197.83 and the ligand rmsd from the input structures revealed 83.17 (Table 2).

### 3.5. Experimental Validation of the NOS Gene in TV

**Figure 6:**
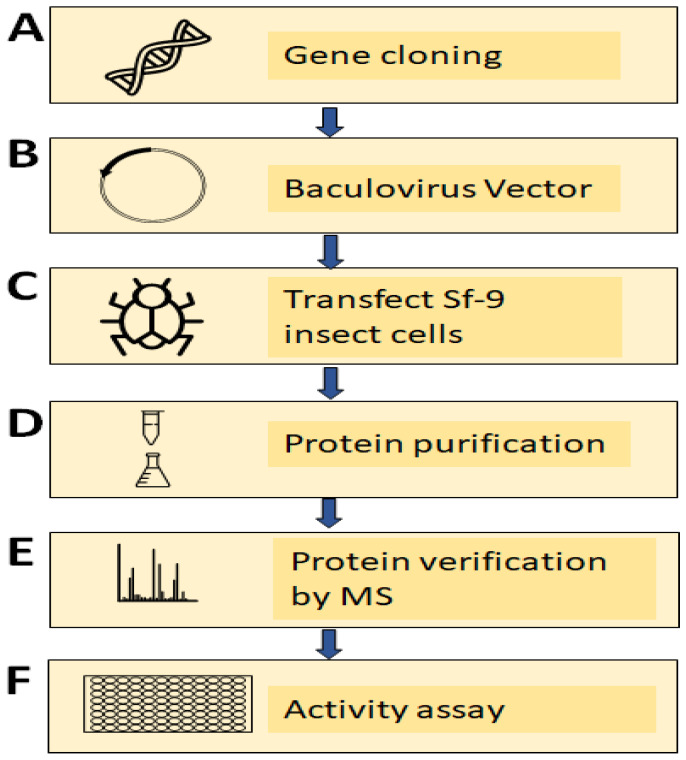
Diagram illustrating the serial experimental steps to validate TVAG_136330 as a nitric oxide synthase in TV, including (**A**) cloning of TVAG_136330 by using a PCR-based accurate synthesis method, (**B**) subcloning of TVAG_136330 to the baculoviral vector, (**C**) transfection of sf-9 insect cells with the baculoviral vector to generate virus, (**D**) transduction of insect cells with viruses to overexpress proteins and performing protein purification, (**E**) verification of protein sequence by mass spectrometry, and (**F**) verification of NOS activity in purified TVAG_136330 proteins.

**Figure 7:**
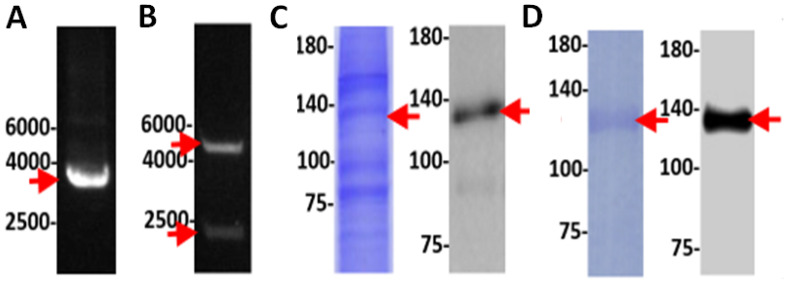
Electrophoresis and staining of biomolecules during the serial steps to clone TVAG_136330 gene and express TVAG_136330 proteins including (**A**) intact plasmids with gene insert, (**B**) digested plasmids showing separated plasmid backbone and gene insert, (**C**) a crude protein sample (left) with antibody staining of TV NOS (right), and (**D**) purified protein sample (left) with antibody staining of TV NOS (right). Note that most impurities in protein were removed after purification.

**Figure 8:**
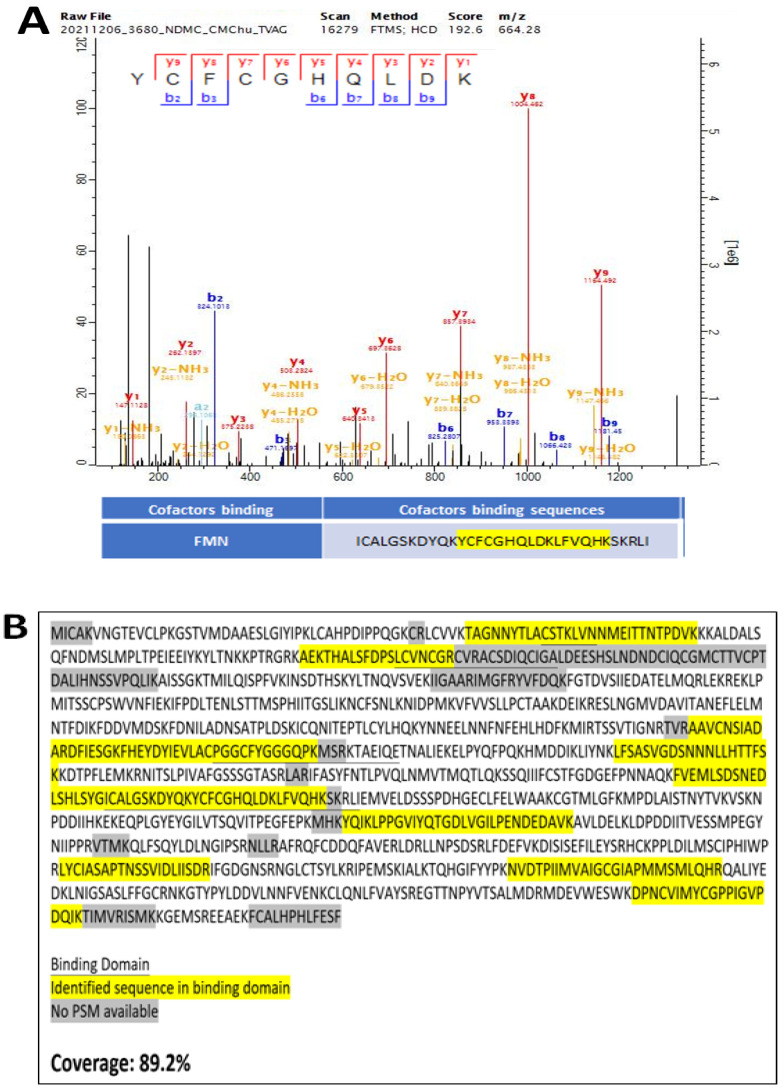
Mass-spectrometry-based sequence analysis of purified TVAG_136330 proteins. Both (**A**) peptide data with daughter ions which match the trypsin-digested FMN-binding sites (YCFCGHQLDK) and (**B**) a high sequence coverage which occupies 88.2% of protein length and includes all cofactor-binding sites indicate that the correct protein was successfully cloned and expressed.

**Figure 9:**
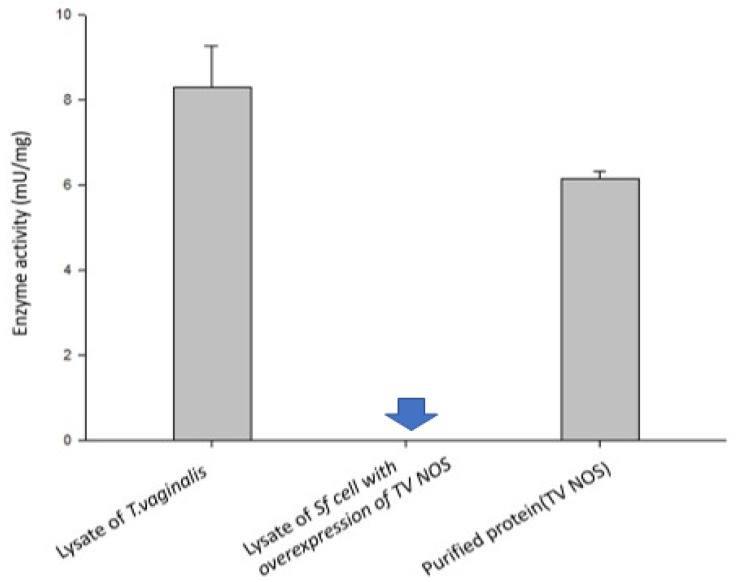
Analysis of NOS activities in crude lysate from TV cells, in crude lysate from Sf insect cells exogenously overexpressing TV NOS and with purified TV NOS proteins. Note that activity of TV NOS (blue arrow, mean = 0.009, SD: 0.002) was greatly suppressed in Sf cells as compared to TV cells (mean = 8.2970, SD: 0.966) and purified TV NOS proteins (mean = 6.160, SD: 0.176), suggesting that certain NOS inhibitor exists in insect cells which can block NOS activity.

To validate the putative NOS in TV, the TV NOS genes were cloned by using a bac-to-bac protein expression system (Figure 6, Figure 7 and Figure 8). Purification of TV NOS protein is shown in Figure 7. To confirm protein identification, liquid chromatography-tandem mass spectrometry (LC–MS/MS) was conducted using in-gel digestion and in-solution digestion. The results of LC–MS/MS and the analysis with species-specific amino acid sequences revealed a coverage of 89% for TV NOS (Figure 7, Supplementary Materials and Methods, Figures S12–S19 and Tables S1 and S2). The FMN polypeptide sequence fragments covered in the LC–MS/MS data searched in NCBI BLASTp showed 100% identity and 100% positives to the TV NOS protein sequence (gene name: TVAG_136330). The sequence search analysis by a Mascot search revealed top scores of 30288 and 15708 with accession numbers XP_001580488.1 and the UniProt database of reviewed Spodoptera frugiperda proteins (TVAG revealed in-solution digestion and TV120 revealed in-gel digestion for 120 kDa, respectively) (Table S1). These results confirm the presence of a TV NOS protein. The NOS activity was detected by using the NOS Activity Assay Kit and is shown in Figure 9. The results of the crude lysate of TV protein and TV NOS protein revealed enzyme activities of 8.2970 and 6.160 (mu/mg), respectively, showing that both TV and TV NOS protein had NOS activities. However, activity of TV NOS (mean = 0.009, SD: 0.002) was greatly suppressed in Sf cells as compared to TV cells (mean = 8.2970, SD: 0.966) and purified TV NOS proteins (mean = 6.160, SD: 0.176), suggesting that a certain NOS inhibitor exists in insect cells, which can block NOS activity (Figure 9).

## 4. Discussion

The gene annotation of NOS in TV is now supported by our witness-to-suspect hybrid bioinformatic annotation method and verified by experimental assay. In addition, we found a novel NOS gene (TV NOS) that is compatible with Harris’s proposal.

In this study, we also compared the TV NOS amino acid sequence with the top three highest scores of NOS amino acid sequences and the top three rank sequences in the Giardia genome program (“GiardiaDB,” 2012) using BLAST provided by NCBI and MATLAB software for multiple alignment. Many studies have revealed that NOS in different species has an oxygenase domain as its central axis and many other studies have shown that NOS in both evolutionarily classified prokaryotic bacteria and eukaryotic mammals has an oxygenase domain [9]. However, the three Giardia NOS that are evolutionarily similar to TV have no oxygenase domain and the conserved domains of the three domains in Giardia were included in our two target sequences.

This study also extends the species of the top three highest rank NOS sequences compared to the target sequence. Compared with TV NOS, the highest scores of the three NOS sequences are sequentially predicted to be the NOS sequences of the *Aspergilllus flavus*, the mammalian *Rattus norvegicus*, and the insect *Lucidina biplagiata*. Our results showed that TV NOS may be similar to mammalian enzymes, which was comparable to Harris et al.’s proposal [6,21]. That is, the species with high-NOS sequence similarity in comparison with our target sequence are not restricted to prokaryotes or eukaryotes.

The annotation method in this study was to use the cross-species NOS sequence to identify the presence or absence of the target protein in TV. We used the MUSCLE multiple-sequence alignment tool to perform multiple alignment comparisons of TV NOS with three kinds of human NOS. The results show that the identity of TV NOS and the three NOS is between 23 and 27% and the positive (or similarity) is approximately 55–57%. Compared with previous studies, *O. tauri* overlaps with human NOS sequences by 44–45% [8,37]. The consistency between *P. polycephalum* and the three animal NOS sequences is less than 39% [17]. That is, TV NOS are less consistent with the sequence of the currently known NOS.

The NOS sequence of TV annotated in this study was found to have a partial structure similar to that of the NOS reductase domain in the NCBI conserved domain search, but it did not have similar oxidation domain features. Although TV NOS did not show a similar oxidative domain in the NCBI conserved domain data, there are still some sequences of iron-containing proteins (Fe2S2) that may replace the original heme protein in the oxidized domain. It is well known that the electron flow in the NOS reaction is NADPH --> FAD --> FMN --> heme --> O(2). TV NOS lacks a mammalian N-terminal domain but contains a flavodoxin domain, a flavin adenine dinucleotide (FAD)-binding domain, and a nicotinamide adenine dinucleotide (NAD)-binding domain structure at the C-terminus, which is comparable to other species. The most interesting feature is that the N-terminal domain contains iron-sulfur proteins (Fe-hydrogenase). Harel et al. [38] reported several ancient connections between two iron-containing redox families (Fe_2_S_2_ and heme-binding domains). These protein families account for half of the metal-containing oxidoreductases that may catalyze redox reactions. By modular evolutionary processes, the heme-binding domains are probably derived from redox reactions, including both anaerobic and aerobic respiration and photosynthesis [38]. Some studies revealed that NO synthesis in *D. radiodurans* could be provided by a surrogate mammalian reductase domain supplied in trans [39]. Wang et al. [40] reported that the flavodoxins of *B. subtilis* support NO production. That is, different types of reductase proteins may support NO production. Similar to the sequence in *Sorangium cellulosum*, TV also had an Fe_2_S_2_ cluster related to electron chemistry in NOS [6,31]. Hunter et al. [41] and Sarkar et al. [39] proposed that *M. phaseolina* possesses an oxygenase domain and that the flavodoxin domain analyzed by InterPro may function as NOS (IPR008524). The result may be comparable to our finding and InterPro also annotates TV flavodoxin/nitric oxide synthase but without experimental validation. In addition, the variation among several species may hinder traditional gene annotation, which can explain some cases of misannotation.

TV NOS was searched against the NOS-related sequences by NCBI BLAST and the results of the first 100 search results were presented as a BLAST TREE. TV NOS is similar to *P. polycephalum* NOS. Using different phylogenetic indicators, it was found that TV is not only similar to prokaryote NOS but may also be similar to an NOS of eubacterial origin or the early evolutionary branches of eukaryotes [42,43].

The rapid progress of artificial intelligence allows us to simulate 2D structures to 3D structures and by means of artificial intelligence, Alphafold2, so that we were able to predict the oxygenase domains that were not predicted by Discovery Studio in the past (Figure S9). The pLDDT and TM score values from the Alphafold2 program are meaningful (Table 1). In addition, the well-known RoseTTAFold artificial intelligence algorithm can also predict the structure of TV NOS and the confidence score is meaningful (Table 1). By using alphafold and RoseTTAFold to predict the protein structure of TV NOS, in silico molecular docking with vital cofactors of NOS (NADPH, BH4, heme, FAD) was performed and revealed good binding affinities. Additionally, docking analysis with the NOS inhibitor (L-NMMA) also showed good binding energy. These results showed various elements of NOS activity in our TV NOS protein.

By simulating two protein 3D structures, we were able to simulate calmodulin binding sites with the HDOCK server to understand protein–protein interactions (Table 2). By using the HDOCK server to investigate the protein–protein interaction of TV NOS protein with calmodulin, the results were found to be relatively consistent with the predicted binding sites between amino acids 513 and 528 (Table 2).

The internal validation results obtained using our hybrid method successfully identified enzymes that were previously experimentally proven. External validation was performed in our lab using previously published NOS-annotated Physarum polycephalum and Ostreococcus tauri strains. By using our annotation method, NOS sequences of both species can also be found. It is proved only by a dry-lab approach without further experimental validation [17,37,44]. In addition, non-TV NOS pie charts consist of many small fragments that show the diversity of species involved in the identification and are not contributed by a single species. In other words, 273 identifications of the non-TV NOS sequences contributed evenly to the TV NOS protein. The TV sequence recognized by non-TV NOS protein sequences presents a concentrated section representing the high identity of the fragments identified by many species, so it is presumed that the two fragments with the higher proportions are the probable NOS sequences of TV [30]. We infer that the candidate sequence criterion can be set by the final witness-to-suspect ratio. When the final witness number is greater than the final number of suspects and the final witness-to-suspect ratio is greater than 30, by using our hybrid annotation method, the verification result is usually valid (Table S3) [30].

Expression of the gene in the bac-to-bac protein expression system has advantages for characterization because it is a eukaryotic protein expression system and TV is also a eukaryotic organism. Kinetic assays confirmed that the recombinant TV NOS enzyme is functional. However, the difficulty in conducting this study was NO production. Wu et al. [36] reported that NO had a cytotoxic effect on SF 21 cells, making the collection of recombinant NOS protein difficult. They proposed that this phenomenon was noted in other recombinant transmembrane proteins [36]. In the past, we also used *E. coli*-competent cells for NOS protein production and cytotoxic effects were also noted in our laboratory of TV NOS and TV2 proteins [38,39]. Furthermore, difficulty in the production of NOS in the bac-to-bac protein expression system was also noted during TV NOS recombinant protein expression in our study. Validation of the TV2 gene by using the bac-to-bac protein expression system in eukaryotic insect cells is for future work.

Although Harris et al. [6] proposed two isoforms of NOS in 2007 (UniProt KB No: Q8 WQT4, A2G9E0), the protein sequences of TV NOS were not similar to their findings. Our findings provide novel biological insights into gene annotation. The discovery of a novel NOS gene in TV has important implications, not only for medical research but also for reducing the burden of costs in scientific studies. Our hybrid gene annotation method can enable the rapid and accurate identification of unknown gene functions in multiple species.

## 5. Conclusions

As there are no previous studies on the functional annotation of TV NOS in this study, our hybrid annotation bioinformatics methods were used to predict the TV NOS sequences (novel TV NOS gene), whose NOS activity was experimentally verified, proving that our annotation method is practical. Further applications may involve the gene function annotation of other species.

## Figures and Tables

**Table 1:** Prediction of the 3D model of TV NOS in RoseTTAFold and AlpaFold2. Both proteins have no close homologs in the Protein Data Bank (PDB).

Protein	RoseTTAFold (Confidence Score)	AlphaFold2 (pLDDT)	AlphaFold2 TM Score
TV NOS	0.82 (all models)	84.05 (model 1)	0.6576 (model 3)

**Table 2:** Prediction of protein–protein interactions by HDOCK of TV NOS.

Protein	Docking Score	Ligand rmsd	Receptor–Ligand Interface Residue Pair
**TV NOS (receptor)**	−197.83	83.17	513–527
**Calmodulin (Ligand)**			

## Data Availability

All the data and materials in the current study are available upon reasonable request.

## Supplementary Materials

The following supporting information can be downloaded at: https://www.mdpi.com/article/10.3390/biology11081210/s1, Figure S1a. NOS-A total of 273 identifications of the non-TV NOS protein sequences for searching candidate proteins. Figure S1b *T. vaginalis*—a total of 6 sequences of the *T. vaginalis* sequence recognized as candidates of NOS. Figure S2. Ligand–receptor interaction results of Mus musculus protein (3DWJ) with heme. Three-dimensional (3D) representation of 3DWJ in complex with heme with the highest binding energy of −10.0 kcal/mol. The accompanying table shows summary results of the analysis; Figure S3. Protein–cofactor interaction results of TV NOS with heme. (**a**) Three-dimensional (3D) representation of TV NOS in complex with heme with the highest binding energy of −7.8 kcal/mol. (**b**) Two-dimensional (2D) representation of TV NOS in complex with heme, showing interactions with three conventional H-bonds, with interactions further stabilized by different amino acids around the TV NOS backbone. The accompanying table shows summary results of the analysis; Figure S4. Protein–cofactor interaction results of TV NOS with BH4. (**a**) Three-dimensional (3D) representation of TV NOS in complex with heme with the highest binding energy of −7.2 kcal/mol. (**b**) Two-dimensional (2D) representation of TV NOS in complex with BH4, showing interactions with three conventional H-bonds, with interactions further stabilized by different amino acids around the TV NOS backbone. The accompanying table shows summary results of the analysis; Figure S5. Protein–cofactor interaction results of TV NOS with FMN. (**a**) Three-dimensional (3D) representation of TV NOS in complex with heme with the highest binding energy of −6.5 kcal/mol. (**b**) Two-dimensional (2D) representation of TV NOS in complex with FMN, showing interactions with four conventional H bonds, with interactions further stabilized by different amino acids around the TV NOS backbone. The accompanying table shows summary results of the analysis; Figure S6. Protein–cofactor interaction results of TV NOS with FAD. (**a**) Three-dimensional (3D) representation of TV NOS in complex with FAD with the highest binding energy of −7.7 kcal/mol. (**b**) Two-dimensional (2D) representation of TV NOS in complex with FAD, showing interactions with ten conventional H bonds, with interactions further stabilized by different amino acids around the TV NOS backbone. The accompanying table shows summary results of the analysis; Figure S7. Protein–cofactor interaction results of TV NOS with NADP. (**a**) Three-dimensional (3D) representation of TV NOS in complex with NADP with the highest binding energy of −8.2 kcal/mol. (**b**) Two-dimensional (2D) representation of TV NOS in complex with NADP, showing interactions with nine conventional H bonds, with interactions further stabilized by different amino acids around the TV NOS backbone. The accompanying table shows summary results of the analysis; Figure S8. Protein–cofactor interaction results of TV NOS with NOS inhibitor (L-NMMA). (**a**) Three-dimensional (3D) representation of TV NOS in complex with L-NMMA with the highest binding energy of −5.6 kcal/mol. (**b**) Two-dimensional (2D) representation of TV NOS in complex with L-NMMA, showing interactions with seven conventional H bonds, with interactions further stabilized by different amino acids around the TV NOS backbone. The accompanying table shows summary results of the analysis; Figure S9. pFastBac1 Vector of TV NOS; Figure S10. Simulated models of the TV NOS and TV2 reductase domains by Discovery Studio Alignment of sequence and structure models. Superposition of the models of the TV NOS reductase domain (**a**) (cyan) and TV2 reductase domain (**b**) (green) with the structure of the rat nNOS reductase domain (PDB code: 1tll) (gray). The backbone root mean square deviation (RMSD) was 8.1 and 6.8 Å for the models of the TV NOS and TV2 reductase domains, respectively; Figure S11. Whole blot images and densitometry readings of TV NOS proteins and his-tag controls. Figure S12. Mass spectrometry protein sequence of the heme binding site; Figure S13. Mass spectrometry protein sequence of the BH4 binding site; Figure S14. Mass spectrometry protein sequence of the calmodulin binding site; Figure S15. Mass spectrometry protein sequence of the FMN binding site; Figure S16. Mass spectrometry protein sequence of the FAD pyrophosphate binding site; Figure S17. Mass spectrometry protein sequence of FAD isoaaloxazine binding site; Figure S18. Mass spectrometry protein sequence of NADPH ribose binding site; Figure S19. Mass spectrometry protein sequence of the NADPH adenine binding site; Figure S20. Internal validation and external validation of this bioinformatic method in our laboratory [6]; Table S1. Protein list of LC/MS/MS identification in Spodoptera frugiperda and the TVAG database (top 10 for each sample); Table S2. NOS cofactor binding sites, sequences and TV NOS mass spectrometry protein sequences, including NCBI BLASTp results (including identity and positives); Table S3. The summary of the ratio of i/j and i/30 in the annotation process in our laboratory. Figure S21. The result of protein sequences of TV NOS submitted to INTERPRO website. References [45,46,47,48,49] are cited in the Supplementary Materials.

## Abbreviations

TV	*Trichomonas vaginalis* or *T. vaginalis*
NOS	nitric oxide synthase
NGS	next-generation sequencing
NOSoxy	NOS oxygenase domain
NOSred	NOS reductase domain
CaM	calmodulin
bNOS	bacterial NOS
ADH	arginine dihydrolase
ADI	arginine deiminase
IPGs	identical protein groups
FMN	flavine mononucleotide
NADPH	nicotinamide adenine dinucleotide phosphate
BH4	tetrahydrobiopterin
