# Supplementary material for: Innovative Hybrid-Alignment Annotation Method for Bioinformatics Identification and Functional Verification of a Novel Nitric Oxide Synthase in Trichomonas vaginalis
Source: Biology (Basel). 2022 Aug 12;11(8):1210. doi: 10.3390/biology11081210 (PMC9404748; doi:10.3390/biology11081210)
Supplement: Supplementary file 1 [file biology-11-01210-s001.zip › biology-1746307-supplementary.pdf]

Supplementary information

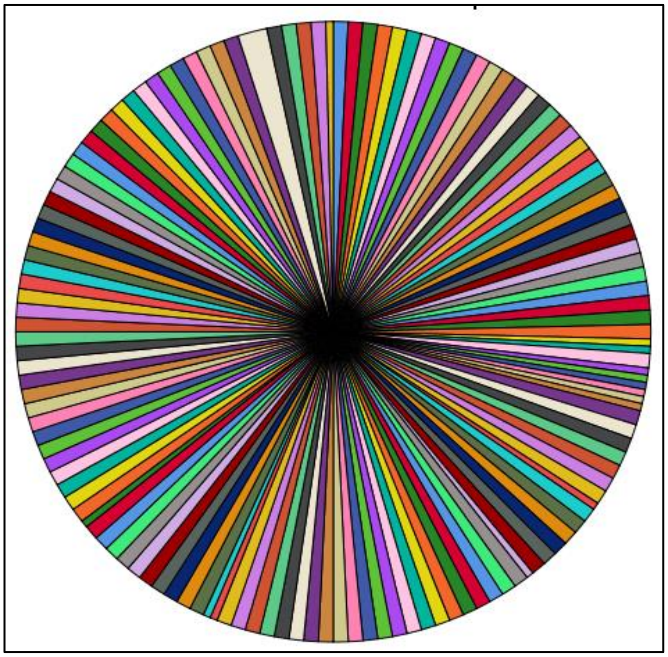

Supplementary Figure S1a. NOS-A total of 273 identifications of the non-TV NOS protein sequences for searching candidate proteins.

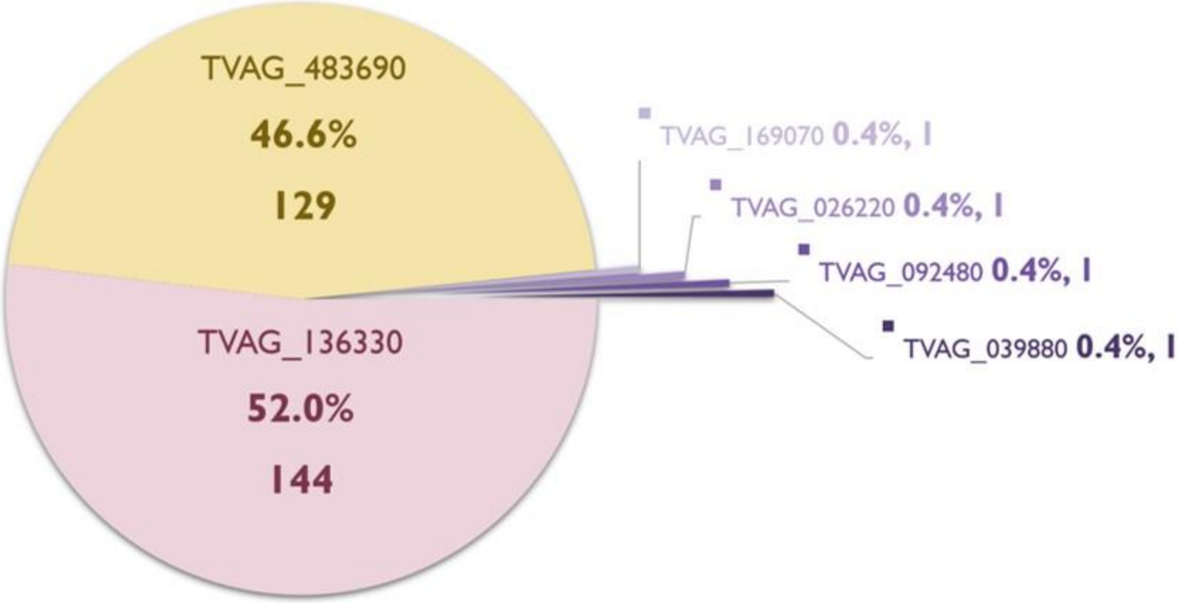

Supplementary Figure S1b *T. vaginalis*- a total of 6 sequences of the *T. vaginalis* sequence recognized as candidates of NOS.

MATLAB Syntax

- Identify repetitive sequences

```
j=fastaread('Folder Path\TV120958.fasta')   %read file

T1=struct2table(j(1:60000))        %T1- Convert the 1st - 60,000th data to table
T1.Properties.RowNames=T1.Header   %Convert the T1 variable Header to rowname
T1.Header=[]

T2=struct2table(j(60001:120958))   %T2- Convert the 60001th -120958th data to table
T2.Properties.RowNames=T2.Header   %Convert the T2 variable Header to rowname
T2.Header=[]

T=[T1;T2]    %T=combine T1&T2
uT=unique(T)   %Identify repetitive sequences

S = table2struct(uT,'ToScalar',true) %Convert table to struct format
S.Header = uT.Properties.RowNames     %Convert rowname to variable

combineS=[S.Header,S.Sequence]        %% Merge the field
colHeadings = {'Header','Sequence'}   % Define the name of the field
jj=cell2struct(combineS, colHeadings, 2)

fastawrite('Folder Path\TV50769.fasta',jj) %%Export FASTA
```

- Loccal alignment: Smith-Waterman alignment

```
NOS=fastaread('Folder Path\NOS141074.fasta')  % Protein sequences

TV=fastaread('Folder Path\TV120958.fasta')  % Species Sequence

for i=1:14107        %Protein sequence count, data splitting calculation

   for j=1:120958   %whole sequences of TV

      try          % When an error sequence is encountered, jump down automatically

      [Localscore a]=swalign(NOS(i).Sequence,TV(j).Sequence) %local alignment

      if Localscore >100       %Those who save more than 100 points

         a = [i j Localscore]

         save 'Folder Path\NOS14107_TV120958.txt' a -ascii -append

      end

   end

   end

end
```

- Global alignment: Needleman–Wunsch alignment

```
for i=[9555 10451 13258 13601 13628 23503]   %Filtered sequence number

   for j=[9555 10451 13258 13601 13628 23503]

   [s align]=nwalign(TV(i).Sequence,TV(j).Sequence)  %global alignment

      a =[i j s]

save 'Folder Path\TV_NW.txt' a -ascii -append

   end

  end
```

- Phylogenic tree

```
NOS=fastaread('C:\Users\b_soph\Downloads\HungChe\NOS14107_uni3639_plusTV1_2.fasta')
```

```
a=[NOS(13) NOS(108) NOS(109) NOS(373) NOS(374) NOS(375) NOS(717) NOS(1951) NOS(1952)
NOS(1953) NOS(1954) NOS(1955) NOS(1957) NOS(1958) NOS(2023) NOS(2112) NOS(2294) NOS(2295)
NOS(2342) NOS(2343) NOS(2379) NOS(2406) NOS(2407) NOS(2461) NOS(2507) NOS(2508) NOS(2509)
NOS(2510) NOS(2610) NOS(2957) NOS(2960) NOS(3482) NOS(3640) NOS(3641)]

tree = seqlinkage(seqpdist(a),'single', a)

view(tree)
```

Autodock FR simulation

We selected known identified NOS structures to simulate heme binding Mus musculus

(Protein name on Protein Data Bank (PDB): 3DWJ)

The first group of models

mode	Affinity (kcal/mol)	clust. rmsd	ref. rmsd	clust. size	Rmsd stdv	Energy stdv	Best Run
1	-10.0	0	-1.0	4	0.6	0.6	5
2	-8.8	7.8	-1.0	3	0.5	0.2	9
3	-8.1	4.7	-1.0	1	NA	NA	1
4	-7.7	5.9	-1.0	2	0.7	0	10

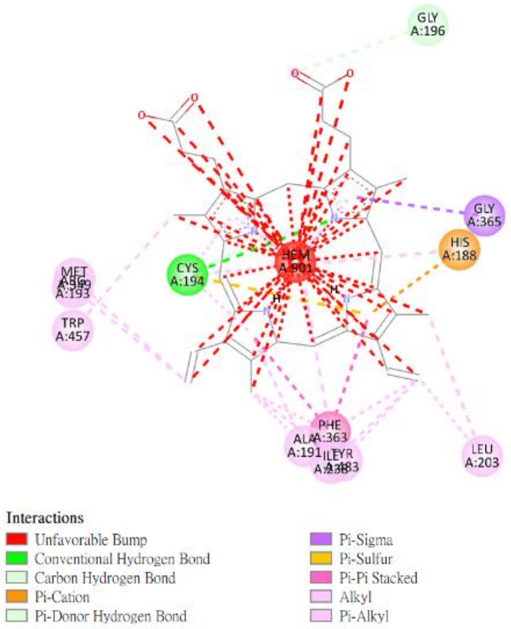

Figure S2. Ligand–receptor interaction results of Mus musculus protein (3DWJ) with Heme. Three-dimensional (3D) representation of 3DWJ in complex with Heme with the highest binding energy of – 10.0 kcal/mol. The accompanying table shows summary results of the analysis.

Schematic diagram of TV1 and TV2 protein simulation

TV1

HEME

Group 1 Model

mode	Affinity (kcal/mol)	clust. rmsd	ref. rmsd	clust. size	Rmsd stdv	Energy stdv	Best run
1	-7.8	0.0	-1.0	3	0.4	0.4	9
2	-7.4	12	-1.0	3	0.4	0.9	6
3	-6.6	4.8	-1.0	3	0.8	0.0	2
4	-5.9	5.5	-1.0	1	NA	NA	8

Close to the predicted position of the 134th amino acid

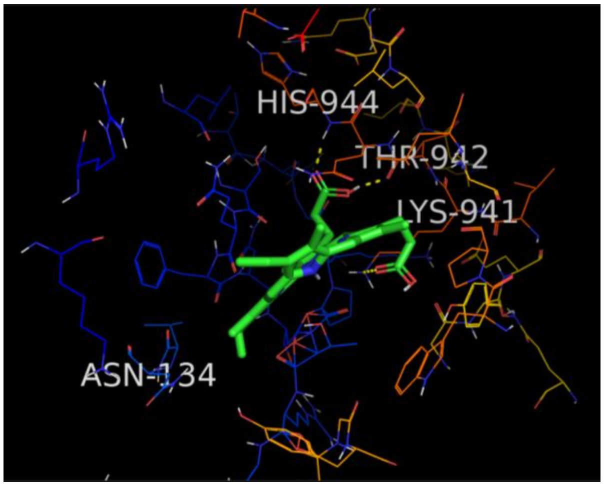

(a)

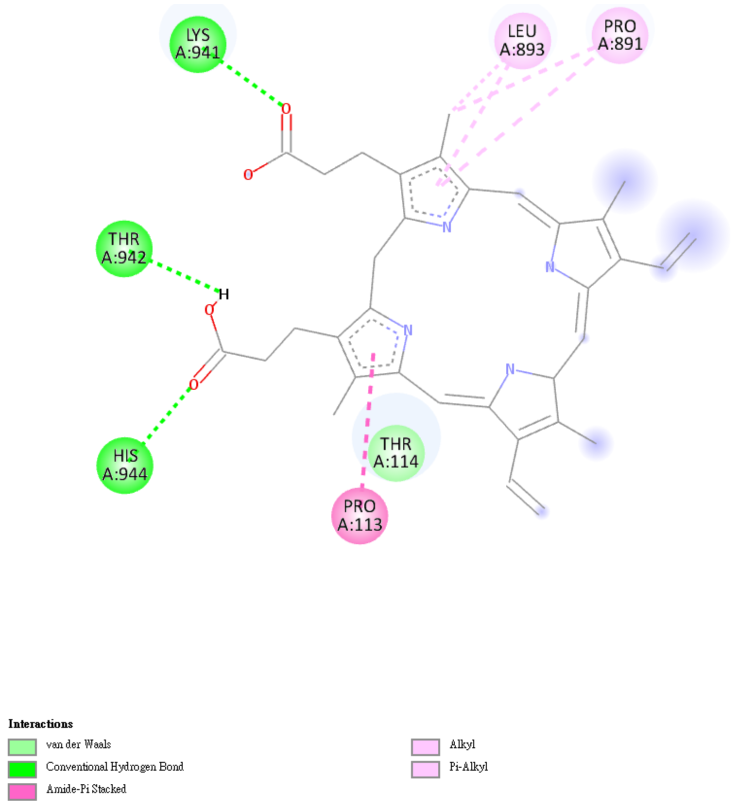

(b)

Hydrogen bond location:

Amino acid	Amino acid position in sequence
LYS	941
THR	942
HIS	944

Figure S3. Protein-cofactor interaction results of TV1 with Heme. (a) Three-dimensional (3D) representation of TV1 in complex with Heme with the highest binding energy of –7.8 kcal/mol.

(b) Two-dimensional (2D) representation of TV1 in complex with Heme, showing interactions with three conventional H-bonds, with interactions further stabilized by

different amino acids around the TV1 backbone. The accompanying table shows summary results of the analysis.

TV1

BH4

Group 3 Model

mode	Affinity (kcal/mol)	clust. rmsd	ref. rmsd	clust. size	Rmsd stdv	Energy stdv	Best run
1	-7.2	0.0	-1.0	8	0.7	0.2	10
2	-7.0	2.7	-1.0	1	NA	NA	8
3	-6.5	2.1	-1.0	1	NA	NA	7

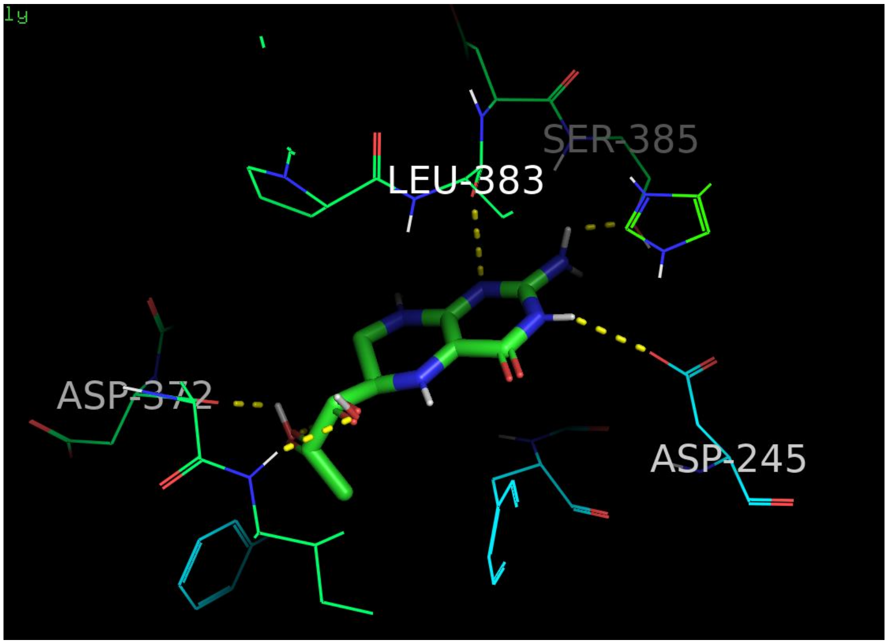

(a)

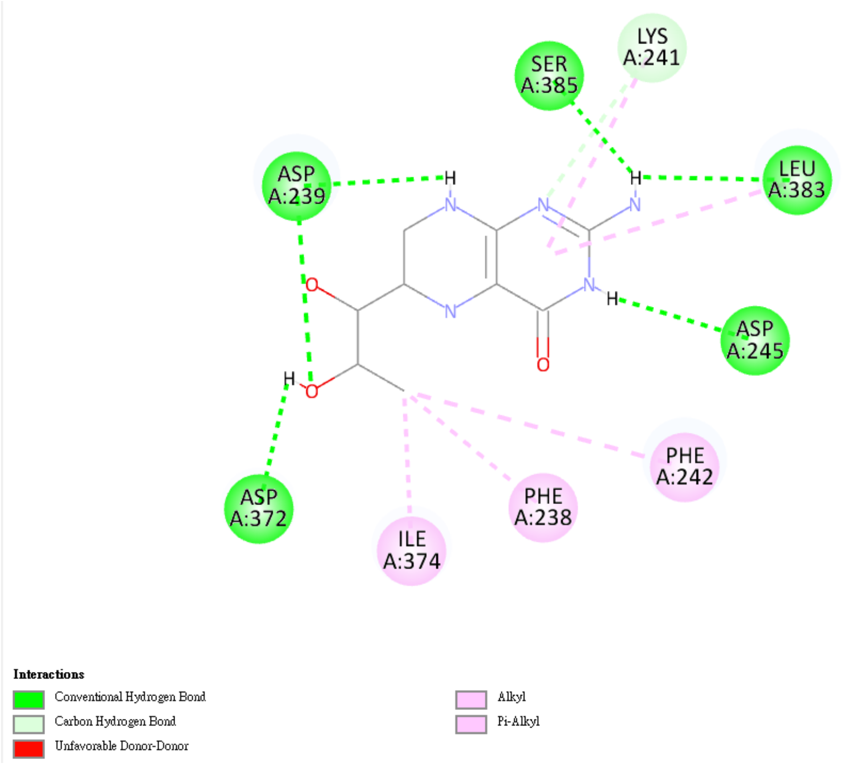

(b)

Hydrogen bond location:

Amino acid	Amino acid position in sequence
ASP	239
ASP	245
ASP	372
ILE	374
LEU	383
SER	385

Figure S4. Protein-cofactor interaction results of TV1 with BH4. (a) Three-dimensional (3D) representation of TV1 in complex with Heme with the highest binding energy of –7.2 kcal/mol.

(b) Two-dimensional (2D) representation of TV1 in complex with BH4, showing interactions with three conventional H-bonds, with interactions further stabilized by different amino acids around the TV1 backbone. The accompanying table shows summary results of the analysis.

TV1

FMN

Group XI Model

mode	Affinity (kcal/mol)	clust. rmsd	ref. rmsd	clust. size	Rmsd stdv	Energy stdv	Best run
1	-6.5	0	-1.0	1	NA	NA	4
2	-6.3	5.5	-1.0	2	0.3	0	2
3	-5.6	5.3	-1.0	3	0.8	0	5
4	-5.6	3.4	-1.0	1	NA	NA	1
5	-5.3	5.2	-1.0	1	NA	NA	8
6	-4.9	3.6	-1.0	1	NA	0.0	7
7	-3.7	4.6	-1.0	1	NA	NA	3

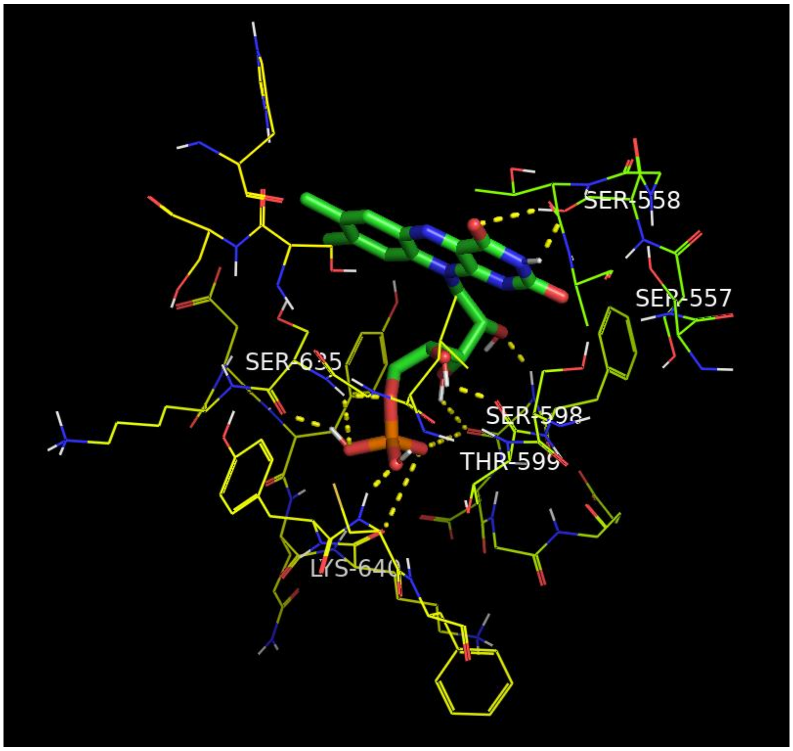
SER-558
SER-557
SER-635
SER-598
THR-599
LYS-640

(a)

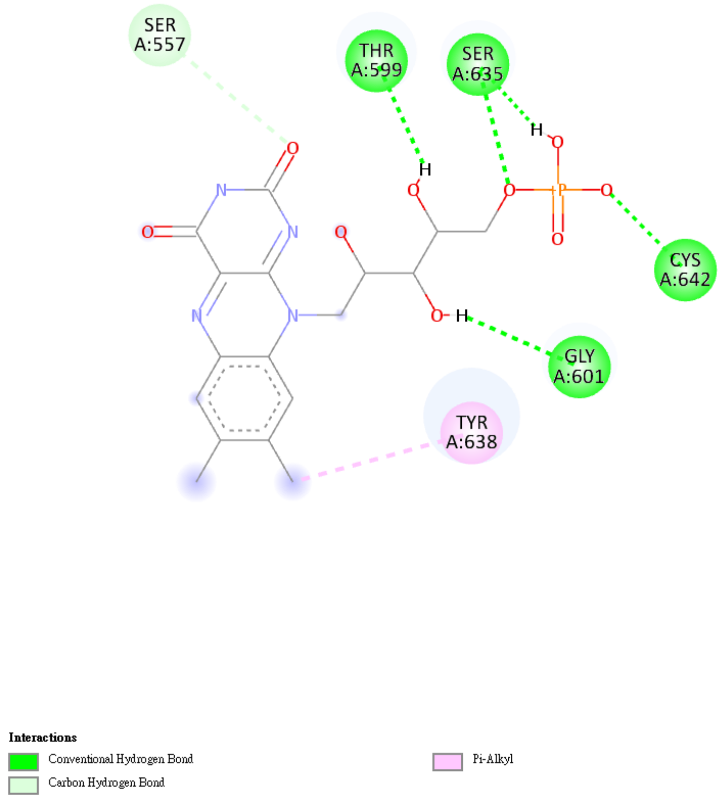
SER A:557
THR A:599
SER A:635
CYS A:642
GLY A:601
TYR A:638
Interactions
Conventional Hydrogen Bond
Carbon Hydrogen Bond
Pi-Alkyl

(b)

Hydrogen bond location:

Amino acid	Amino acid position in sequence
SER	557
THR	599
GLY	601
SER	635
TYR	638
CYS	642

Figure S5. Protein-cofactor interaction results of TV1 with FMN. (a) Three-dimensional (3D) representation of TV1 in complex with Heme with the highest binding energy of –6.5 kcal/mol.

(b) Two-dimensional (2D) representation of TV1 in complex with FMN, showing interactions with four conventional H-bonds, with interactions further stabilized by different amino acids around the TV1 backbone. The accompanying table shows summary results of the analysis.

TV1

FAD

Group I Model

mode	Affinity (kcal/mol)	clust. rmsd	ref. rmsd	clust. size	Rmsd stdv	Energy stdv	Best run
1	-10.6	0.0	-1.0	1	NA	NA	1
2	-9.3	6.1	-1.0	1	NA	NA	9
3	-9.2	4.4	-1.0	1	NA	NA	8
4	-8.4	4.4	-1.0	1	NA	NA	5
5	-8.3	3.2	-1.0	1	NA	NA	4

6	-6.8	9.5	-1.0	2	0.7	0.0	2
7	-6.6	5.9	-1.0	1	NA	NA	7
8	-4.9	9.9	-1.0	1	NA	NA	10
9	-3.5	9.9	-1.0	1	NA	NA	3

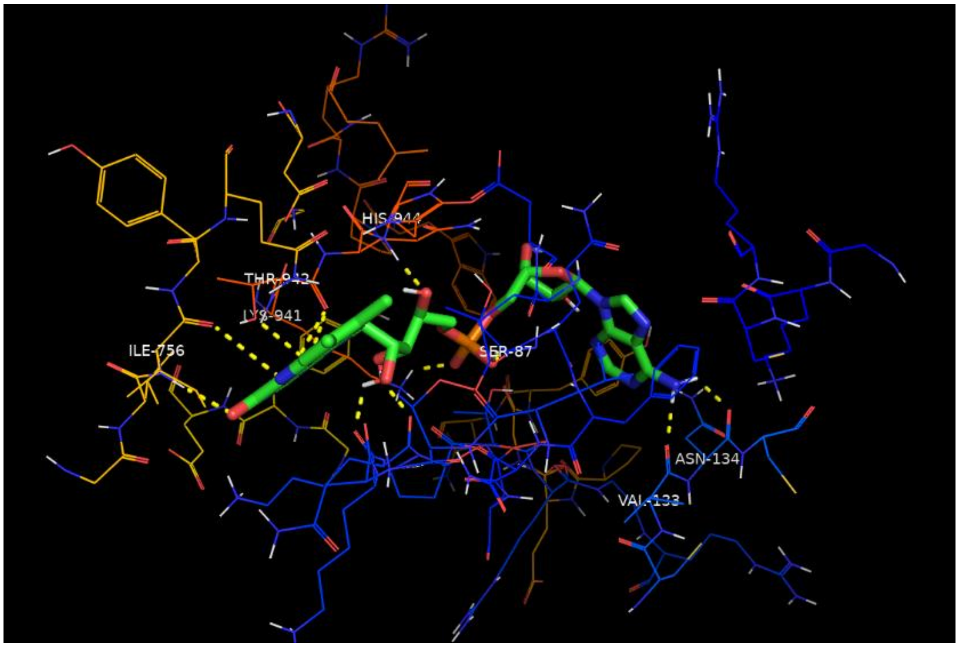

(a)

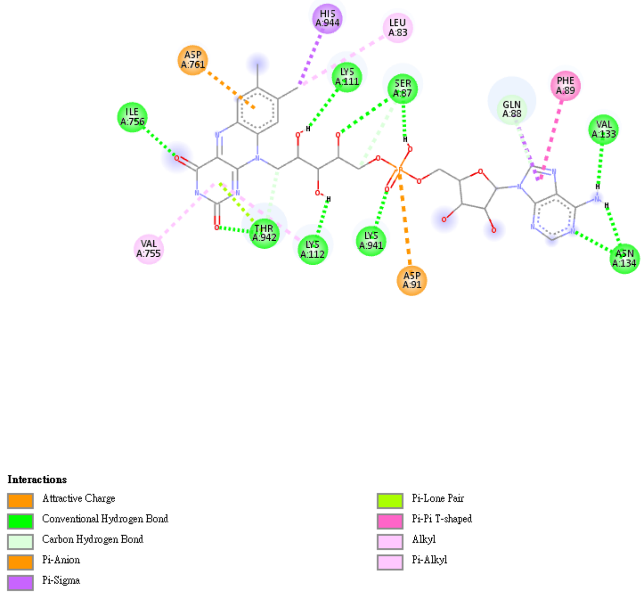

(b)

Hydrogen bond location:

Amino acid	Amino acid position in sequence
SER	87
VAL	133
ASN	134
ILE	756
LYS	941
THR	943
HIS	944

Figure S6. Protein-cofactor interaction results of TV1 with FAD. (a) Three-dimensional (3D) representation of TV1 in complex with FAD with the highest binding energy of –7.7 kcal/mol.

(b) Two-dimensional (2D) representation of TV1 in complex with FAD, showing interactions with ten conventional H-bonds, with interactions further stabilized by different amino acids around the TV1 backbone. The accompanying table shows summary results of the analysis.

TV1

NADP

Group V Model

mode	Affinity (kcal/mol)	clust. rmsd	ref. rmsd	clust. size	Rmsd stdv	Energy stdv	Best run
1	-8.2	0.0	-1.0	1	NA	NA	9
2	-6.4	7.7	-1.0	1	NA	NA	1
3	-5.8	7.6	-1.0	1	NA	NA	6
4	-5.0	7.0	-1.0	1	NA	NA	7

5	-5.0	8.6	-1.0	1	NA	NA	4
6	-4.8	6.6	-1.0	1	NA	NA	8
7	-3.6	7.2	-1.0	1	NA	NA	3
8	-3.5	7.9	-1.0	1	NA	NA	2
9	-2.0	6.7	-1.0	1	NA	NA	10
10	0	10.1	-1.0	1	NA	NA	5

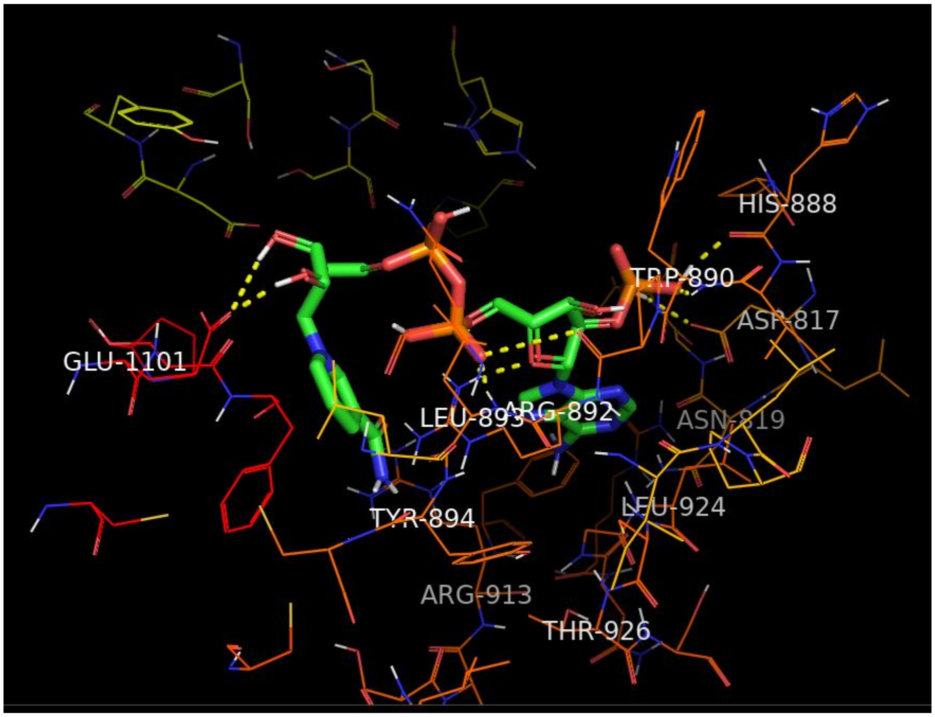

(a)

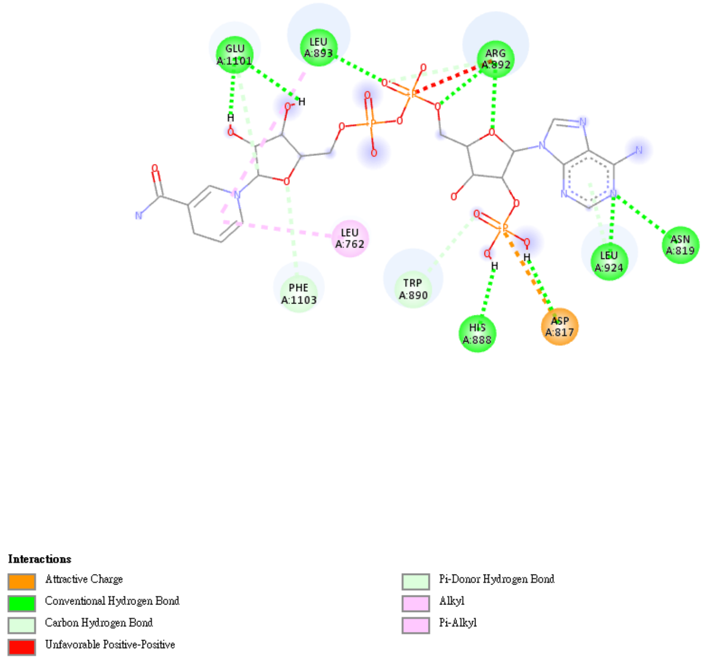

(b)

Hydrogen bond location:

Amino acid	Amino acid position in sequence
ASP	817
ASN	819
HIS	888
ARP	890
ARG	892
LEU	893
LEU	924
GLU	1101
PHE	1103

Figure S7. Protein-cofactor interaction results of TV1 with NADP. (a) Three-dimensional (3D) representation of TV1 in complex with NADP with the highest binding energy of – 8.2 kcal/mol.

(b) Two-dimensional (2D) representation of TV1 in complex with NADP, showing interactions with nine conventional H-bonds, with interactions further stabilized by different amino acids around the TV1 backbone. The accompanying table shows summary results of the analysis.

TV1

L-NMMA

Group 1 Model

Mode	Affinity (kcal/mol)	clust. rmsd	ref. rmsd	clust. size	Rmsd stdv	Energy stdv	Best run
1	-5.6	0	-1.0	2	0.6	0	1
2	-5.4	6.1	-1.0	2	0.3	0	10
3	-5.4	3.2	-1.0	2	0.1	0	3
4	-4.8	6.5	-1.0	2	0.3	0	8
	-4.5	5.1	-1.0	2	0.6	0	9

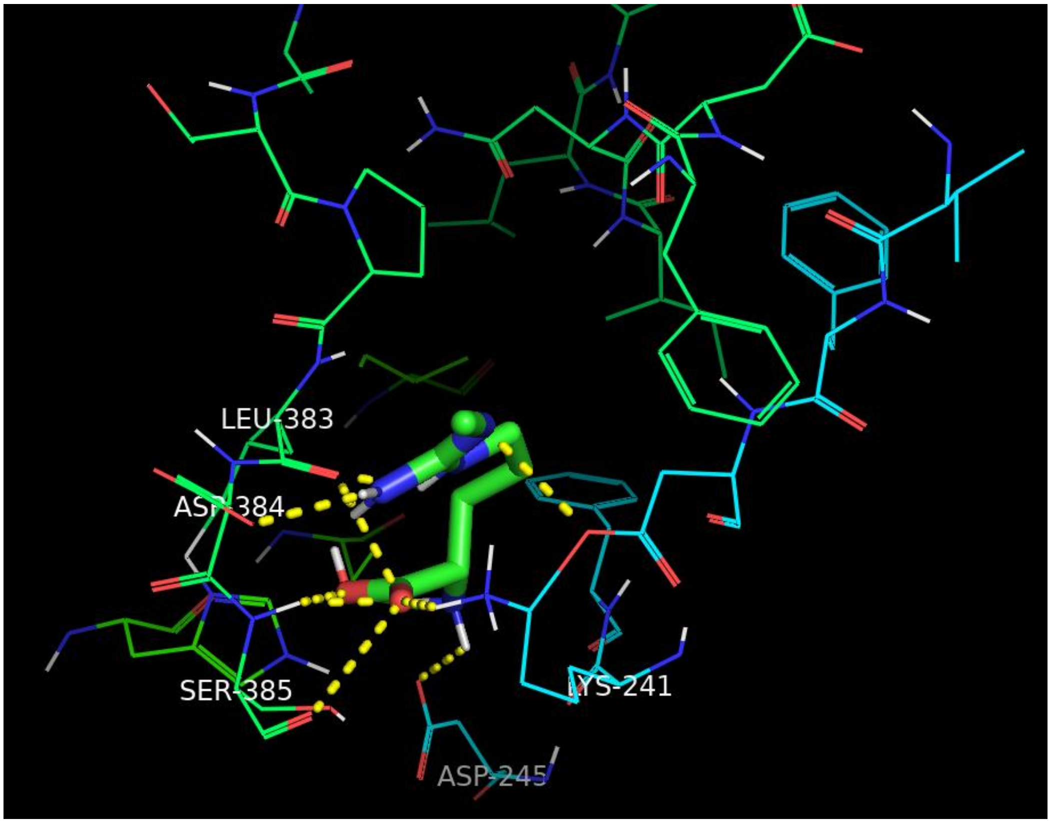
LEU-383
ASP-384
SER-385
LYS-241
ASP-245

(a)

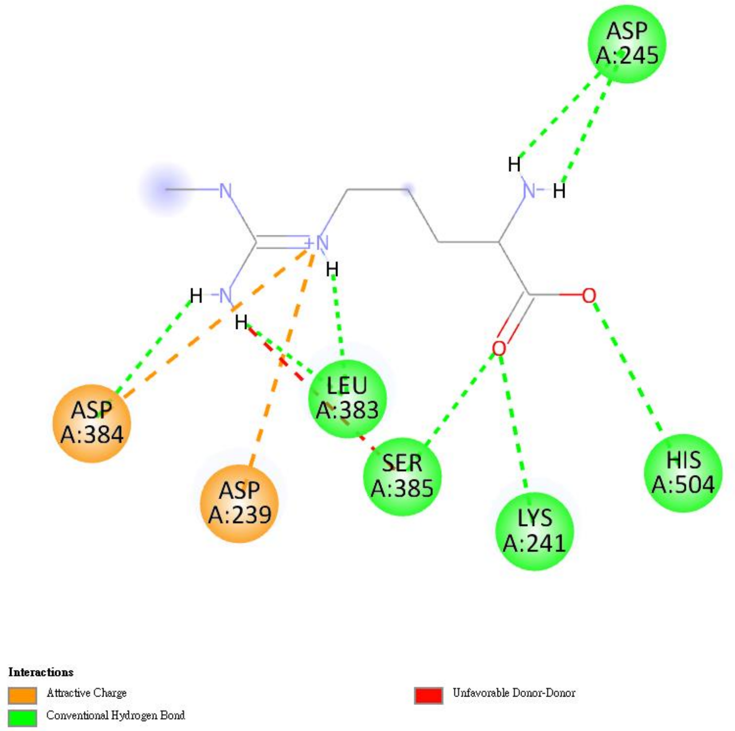
ASP A:245
LEU A:383
ASP A:384
ASP A:239
SER A:385
LYS A:241
HIS A:504
Interactions
Attractive Charge
Conventional Hydrogen Bond
Unfavorable Donor-Donor

(b)

Hydrogen bond location:

Amino acid	Amino acid position in sequence
LYS	241
ASP	245
LEU	383
ASP	384
SER	385
HIS	504

Figure S8. Protein-cofactor interaction results of TV1 with NOS inhibitor(L-NMMA). (a) Three-dimensional (3D) representation of TV1in complex with L-NMMA with the highest binding energy of – 5.6 kcal/mol. (b) Two-dimensional (2D) representation of TV1 in complex with L-NMMA, showing interactions with seven conventional H-bonds, with interactions further stabilized by different amino acids around the TV1 backbone. The accompanying table shows summary results of the analysis.

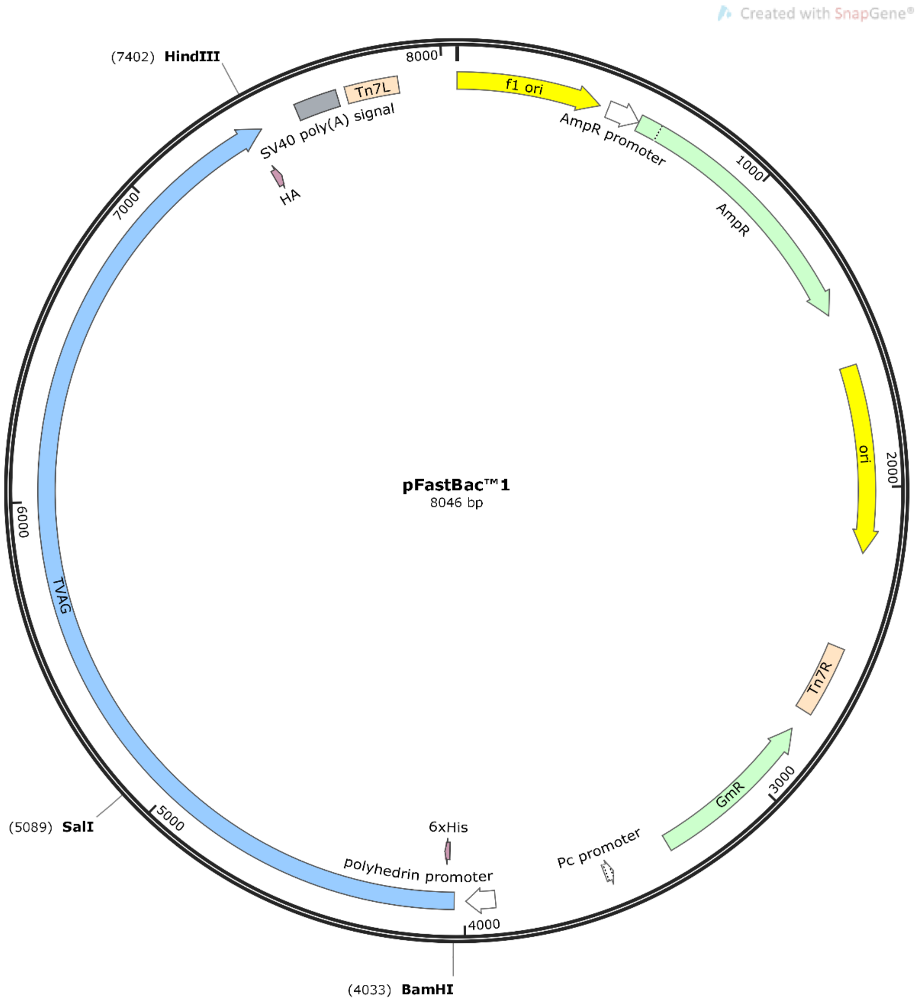

Figure S9. pFastBac1 Vector of TV1.

Simulation of TV1 and TV2 reductase domains by Discovery Studio

The structural models of the TV1 and TV2 reductase domains were built using the homology modeling protocol in the Biovia Discovery Studio 2017 program. The resolved structures of NADPH-cytochrome P450 reductase from human, rat and *Saccharomyces cerevisiae* (PDB codes: 3qe2, 1jal and 2bpo, respectively) and the rat nNOS

FAD/NADP domain (PDB code: 1f20) were used as templates. The validity of the models was assessed using the Verify Protein protocol. The model with the best probability density function (PDF) energy and discrete optimized protein energy (DOPE) score was subjected to the CHARM force field for energy minimization.

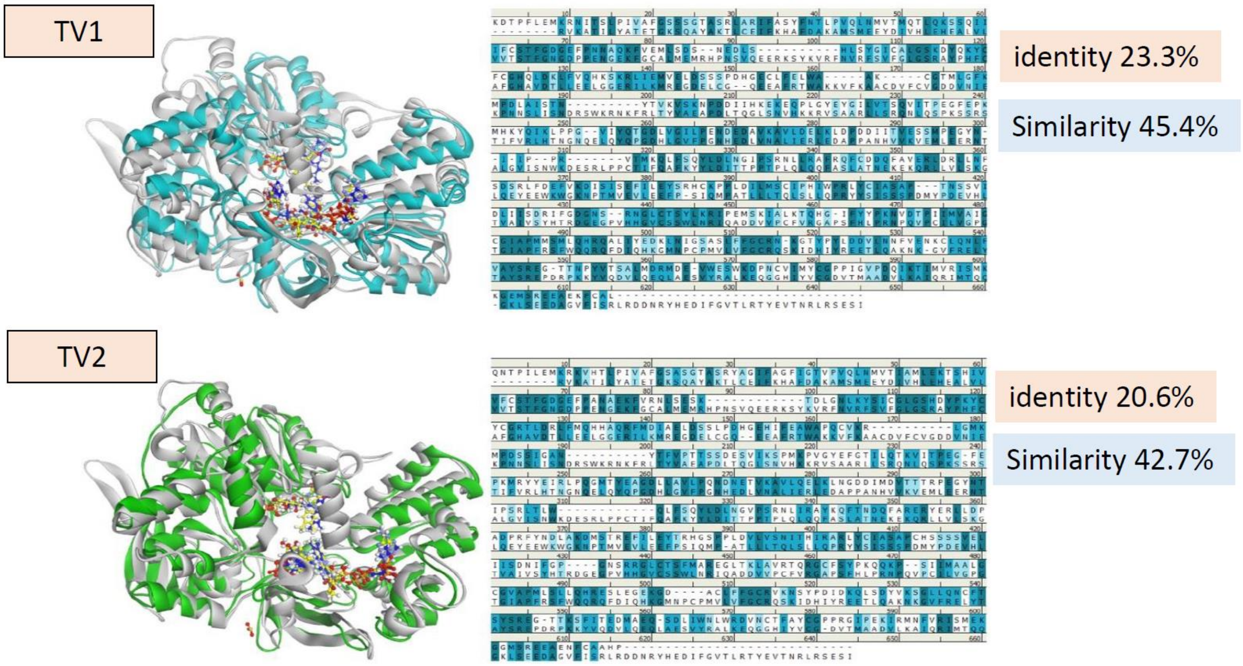

Figure S10. Simulated models of the TV1 and TV2 reductase domains by Discovery Studio

Alignment of sequence and structure models. Superposition of the models of the TV1 reductase domain (a) (cyan) and TV2 reductase domain (b) (green) with the structure of the rat nNOS reductase domain (PDB code: 1tll) (gray). The backbone root mean square deviation (RMSD) was 8.1 and 6.8 Å for the models of the TV1 and TV2 reductase domains, respectively.

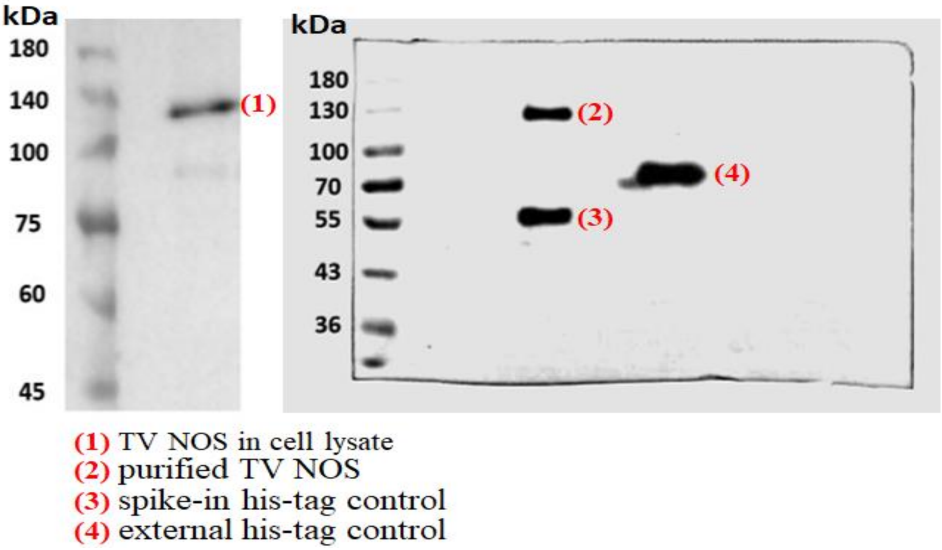

(a)

	**Densitometry readings**
Band (1)	13969
Band (2)	33547
Band (3)	37051
Band (4)	53124
	**Ratios of intensity**
Band (2) / Band (3)	0.905
Band (2) / Band (4)	0.631

(b)

Figure S11. (A) Whole blot images showing TV NOS proteins and his-tag controls. A representative whole blot image of the lysate of SF9 insect cells stained with anti-his tag antibody is displayed, and TV NOS in cell lysate is indicated on the left panel. A representative whole blot image of purified TV NOS together with a spike-in his-tag

control and an external his-tag control loaded in a different lane is displayed on the right panel. **(B)** Densitometry readings of TV NOS in cell lysate and purified TV NOS from the whole blot images are shown. Ratios of purified TV NOS to spike-in his-tag control and external his-tag control are also displayed.

Supplementary materials and methods

LC-MS/MS analysis. Fifty microgram TV NOS protein was dissolved in 6M urea, reduced with 5 mM dithiothreitol for 45 min at 29°C, alkylated with 10 mM iodoacetamide in the dark for 45 min at 29°C, and digested with 1 µg MS-grade Trypsin Gold (Promega, Madison, WI) at 29°C for 16 hr [1-5]. The reaction was terminated by add 10% trifluoroacetic acid (TFA) to a final concentration of 0.5% TFA, and tryptic peptide solution was desalted with C18 ZipTip prior to LC-MS/MS analysis. The eluted peptides were dissolved in 0.1% formic acid, separated using an Ultimate system 3000 nanoLC system (Thermo Fisher Scientific, Bremen, Germany) equipped with a 75 µm ID, 25 cm length C18 Acclaim PepMap NanoLC column (Thermo Fisher Scientific, San Jose, CA, USA) packed with 2 µm particles with a pore of 100 Å. Mobile phase A was 0.1% formic acid in water, and mobile phase B was composed of 100% acetonitrile with 0.1% formic acid. The peptides were eluted at a flow rate of 300 ng/mL with a gradient of 2% to 40% for 90 minutes. Mass spectra were acquired on a Thermo Scientific™

Orbitrap Fusion™ Lumos™ Tribrid™ Mass Spectrometer (Thermo Fisher Scientific, UK). Mass spectrometry analysis was performed in a data-dependent mode with Full-MS (externally calibrated to a mass accuracy of < 5 ppm, and a resolution of 120,000 at *m*/*z* 200) followed by MS/MS analysis of the most intense ions in 3 s. High-energy collision activated dissociation (HCD)-MS/MS (resolution of 15,000) was used to fragment multiply charged ions (Charge state 2-7) within a 1.4 Da isolation window at a normalized collision energy of 32. AGC target at 5e5 and 5e4 was set for MS and MS/MS analysis, respectively with previously selected ions dynamically excluded for 180 s. Max injection time was set as 50 ms. Peptides were identified with MaxQuant software version v2.0.3.1 against TrEMBL Database (*Trichomonas vaginalis* version 2022_03) using Andromeda peptide search engine and the Table S1 was analyzed by using the Mascot searching engine (Proteome Discoverer V2.2.0.388). (TVAG revealed in-solution digestion and TV120 revealed in-gel digestion)

Table S1. Protein list of LC/MS/MS identification in Spodoptera frugiperda and TVAG database. (TOP 10 for each sample)

Sample	Accession No.	Protein Description	Score	MW [kDa]	calc. pI	Coverage [%]
TVAG	XP_001580488.1	Iron only hydrogenase large subunit, C-terminal domain containing protein [Trichomonas vaginalis G3]	30288	123.9	6.02	87
	A0A0K2CTM2	Elongation factor 1-alpha OS=Spodoptera frugiperda OX=7108 GN=SFRICE_011951 PE=2 SV=1	3298	50.3	9.09	76

Sample	Accession No.	Protein Description	Score	MW [kDa]	calc. pI	Coverage [%]
	A0A2H1WFA2	Heat shock 70 kDa protein cognate 4 (Fragment) OS=Spodoptera frugiperda OX=7108 GN=SFRICE003522.2 PE=2 SV=1	2484	71.5	5.47	53
	A0A2H1W8U6	RNA helicase OS=Spodoptera frugiperda OX=7108 GN=SFRICE_014296 PE=3 SV=1	1942	50.4	8.15	75
	A0A2H1WKT6	SFRICE_000817 OS=Spodoptera frugiperda OX=7108 GN=SFRICE_000817 PE=3 SV=1	1749	56.7	9.55	46
	A0A2H1WX92	SFRICE_015077 OS=Spodoptera frugiperda OX=7108 GN=SFRICE_015077 PE=3 SV=1	1722	123.1	6.98	47
	A0A0P0IVE9	Dm0-like lamin OS=Spodoptera frugiperda OX=7108 PE=2 SV=1	1598	70.3	6.68	58
	A0A2H1VJ24	SFRICE_005286 OS=Spodoptera frugiperda OX=7108 GN=SFRICE_005286 PE=3 SV=1	1461	175	7.69	43
	J7ELF8	Elongation factor 1-alpha (Fragment) OS=Spodoptera frugiperda OX=7108 GN=EF1-A PE=4 SV=1	1267	17.5	8.54	65
	A0A2H1WX17	SFRICE016243.2 (Fragment) OS=Spodoptera frugiperda OX=7108 GN=SFRICE016243.2 PE=3 SV=1	1256	73.1	5.34	47
TVAG_120	XP_001580488.1	Iron only hydrogenase large subunit, C-terminal domain containing protein [Trichomonas vaginalis G3]	15708	123.9	6.02	82
	A0A2H1WDM1	SFRICE_000333 OS=Spodoptera frugiperda OX=7108 GN=SFRICE_000333 PE=3 SV=1	977	137.8	6.09	37
	A0A2H1UZY8	SFRICE_003045 OS=Spodoptera frugiperda OX=7108 GN=SFRICE_003045 PE=4 SV=1	599	89.7	6.79	28
	A0A2H1WGM5	Kinesin-like protein OS=Spodoptera frugiperda OX=7108 GN=SFRICE_000564 PE=3 SV=1	595	102.3	5.71	32
	A0A2H1VZQ4	SFRICE_008954 OS=Spodoptera frugiperda OX=7108 GN=SFRICE_008954 PE=4 SV=1	564	129.7	6.71	21
	A0A2P1DMB4	Argonaute 2-PEa OS=Spodoptera frugiperda OX=7108 PE=2 SV=1	559	122.8	9.19	24
	A0A2H1WMH3	SFRICE_015519 OS=Spodoptera frugiperda OX=7108 GN=SFRICE_015519 PE=4 SV=1	558	85.9	8	28

Sample	Accession No.	Protein Description	Score	MW [kDa]	calc. pI	Coverage [%]
	A0A2H1WDQ7	SFRICE_022593 (Fragment) OS=Spodoptera frugiperda OX=7108 GN=SFRICE_022593 PE=4 SV=1	478	70.8	5.54	38
	A0A2H1WKD0	SFRICE_013904 (Fragment) OS=Spodoptera frugiperda OX=7108 GN=SFRICE_013904 PE=4 SV=1	438	138	6.74	18
	A0A2H1VJE7	Ribonuclease Z OS=Spodoptera frugiperda OX=7108 GN=SFRICE_017182 PE=3 SV=1	415	111.6	7.81	23

Criteria: Master protein and score ≥ 25

(TVAG revealed in-solution digestion and TV120 revealed in-gel digestion)

Table S2. NOS Cofactors binding sites, sequences and TV1 Mass Spectrometry protein sequences including NCBI BLASTp results (including identity and positives)

Cofactors binding	Cofactors binding sequences	identity	positives	Mass Spectrometry protein sequence	identity	positives
zinc	CSTKLVN	Multiple species	Multiple species	TAGNNYTLACSTKLVNNME ITTNTPDVK	100	100
heme binding site	LCVNCGRCVRACS DIQCIGA	100	100	AEKTHALSFDPSLCVNCGR	100	100
BH4	PGGCFYGGGQPK MSRKTAEIQE	100	100	AAVCNSIADARDFIESGKFH EYDYIEVLACPGGCFYGGG QPK	100	100
calmodulin	NKLFSASVGDSNN NLL	100	100	LFSASVGDSNNNLLHTTFSK	100	100
FMN	ICALGSKDYQKYC FCGHQLDKLFVQ HKSKRLI	100	100	YCFCGHQLDKLFVQHK	100	100
FAD pyrophosphate	YQTGDLVGILPEN	100	100	YQIKLPPGVIYQTGDLVGILP ENDEDAVK	100	100

FAD isoaaloxazine	IWPRLYCIASA	100	100	LYCIASAPTNSSVIDLIISDR	100	100
NADPH ribose	IMVAIGCGIAPMMS	100	100	NVDTPIIMVAIGCGIAPMMSMLQHR	100	100
NADPH adenine	PNCVIMYCGPPIGVPDQI	100	100	DPNCVIMYCGPPIGVPDQIK	100	100
NADPH	HPHLFESF	Multiple species	Multiple species	none	NA	NA

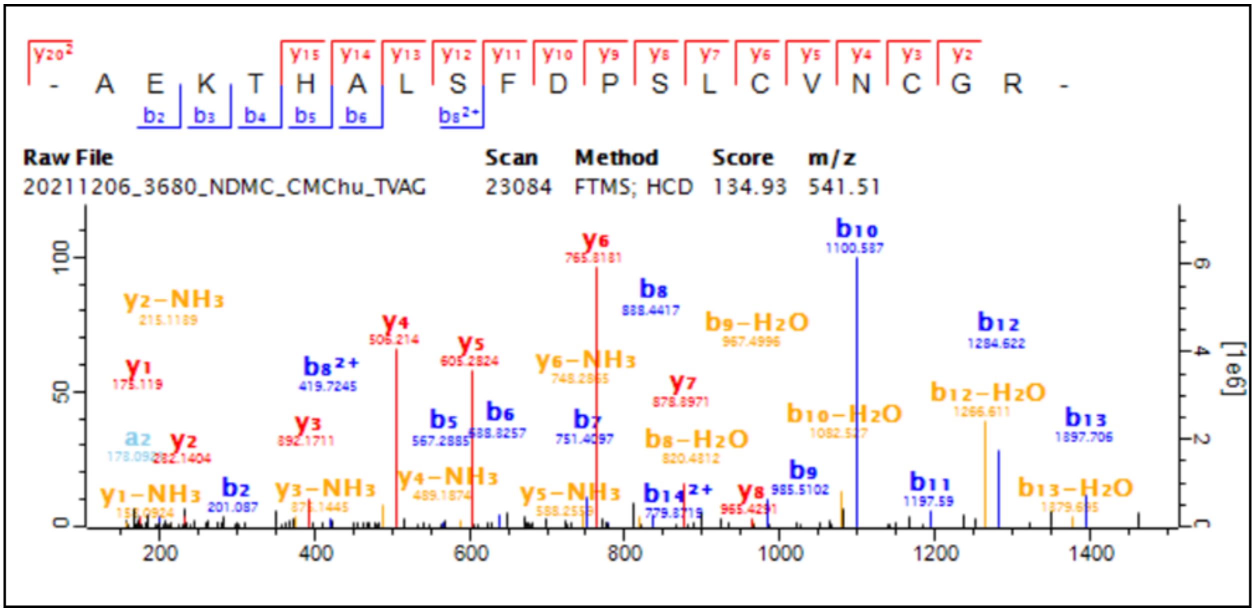

Figure S12. Mass Spectrometry protein sequence of heme binding site

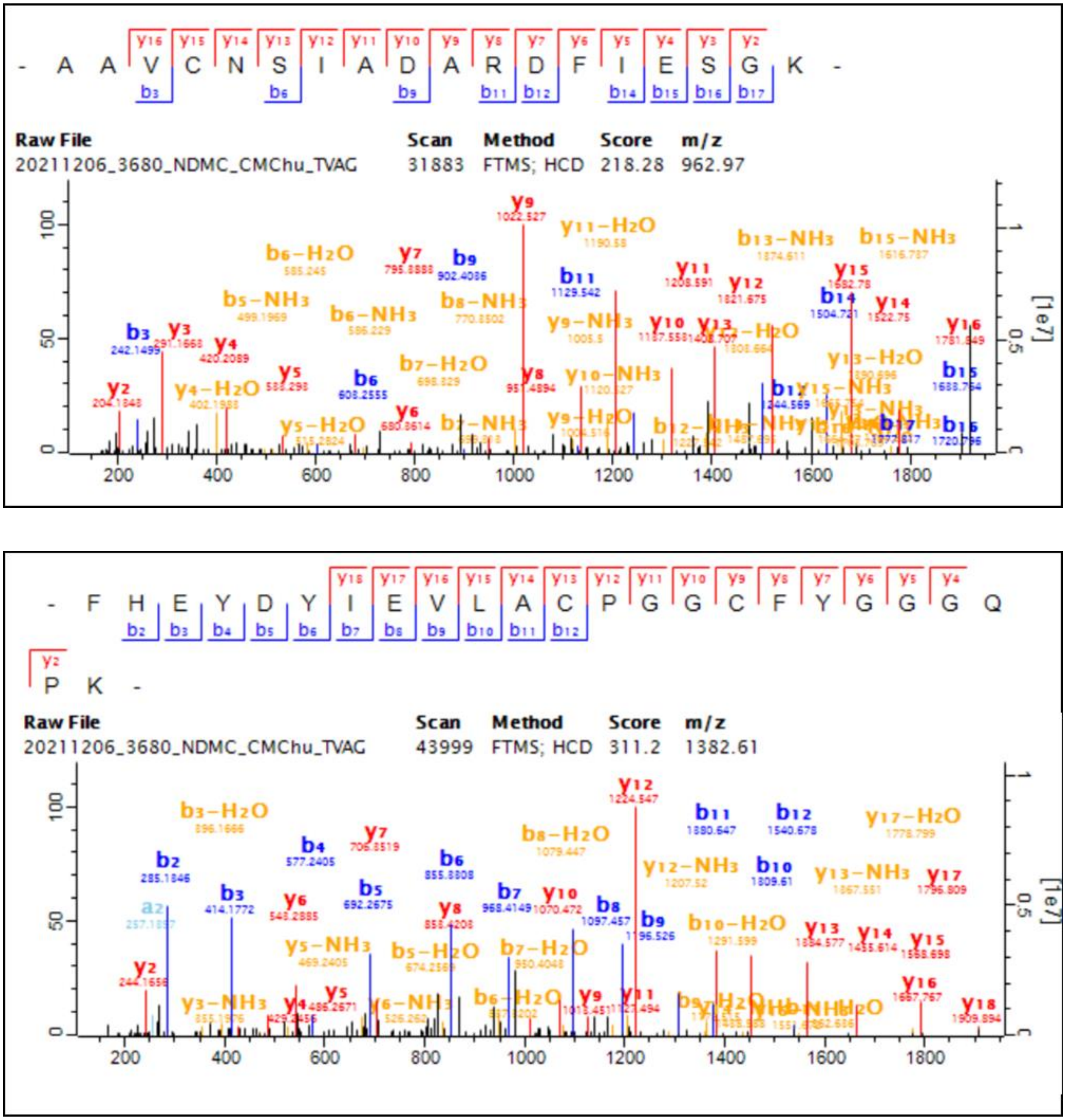

Figure S13. Mass Spectrometry protein sequence of BH4 binding site

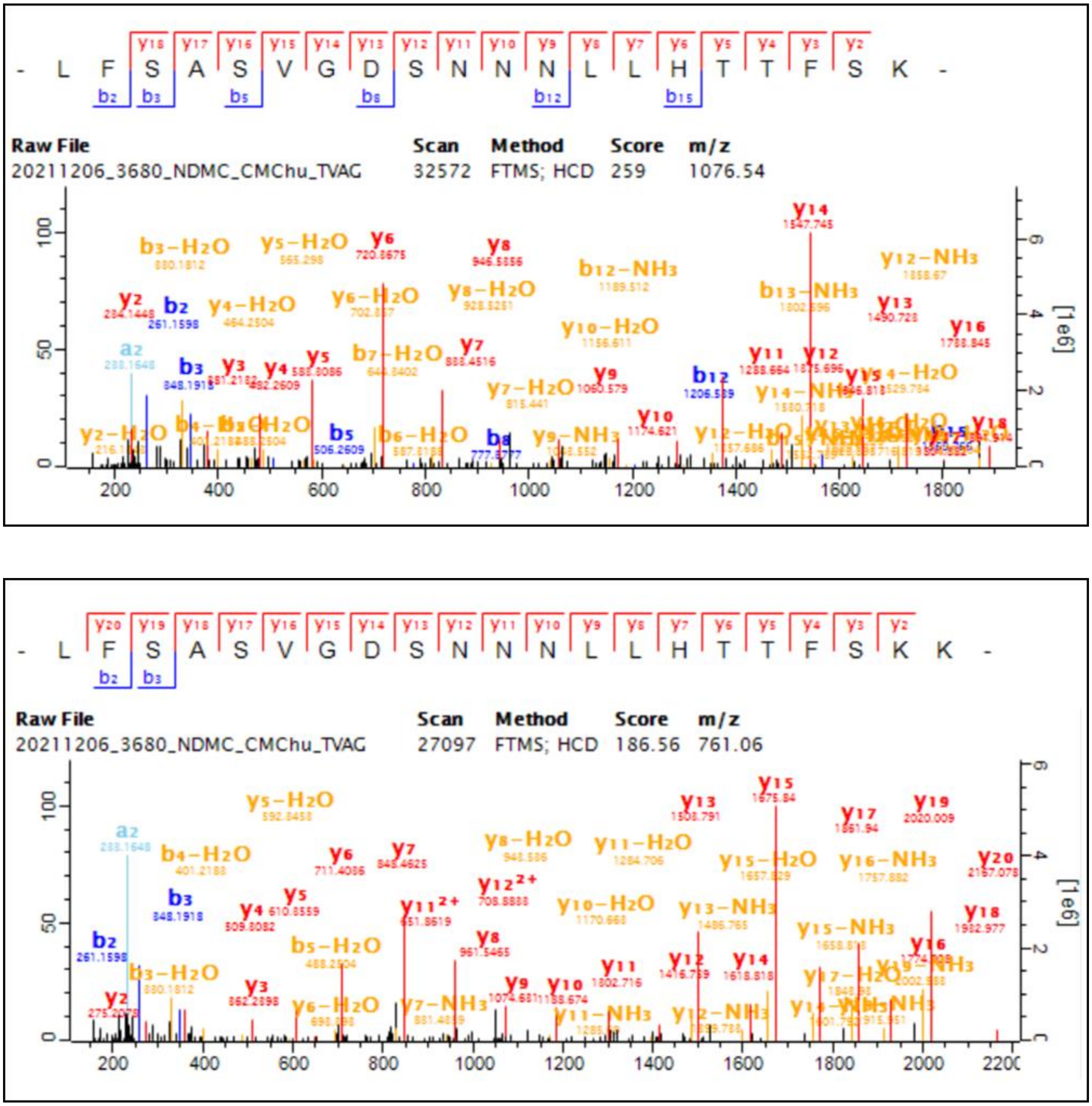

Figure S14. Mass Spectrometry protein sequence of calmodulin binding site

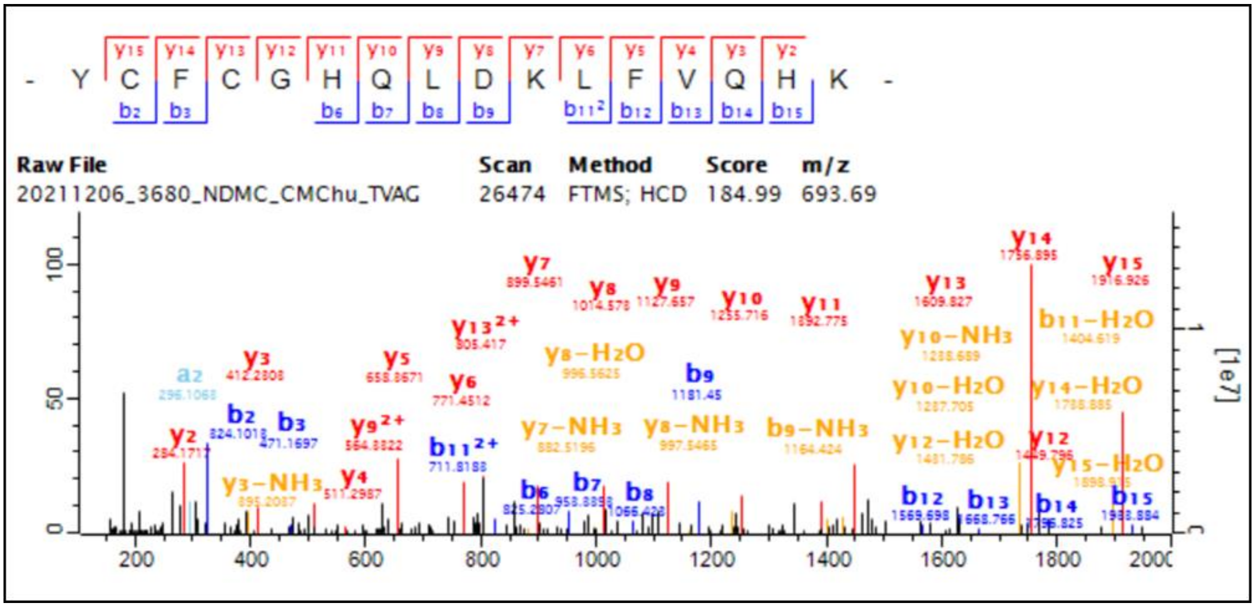

Figure S15. Mass Spectrometry protein sequence of FMN binding site

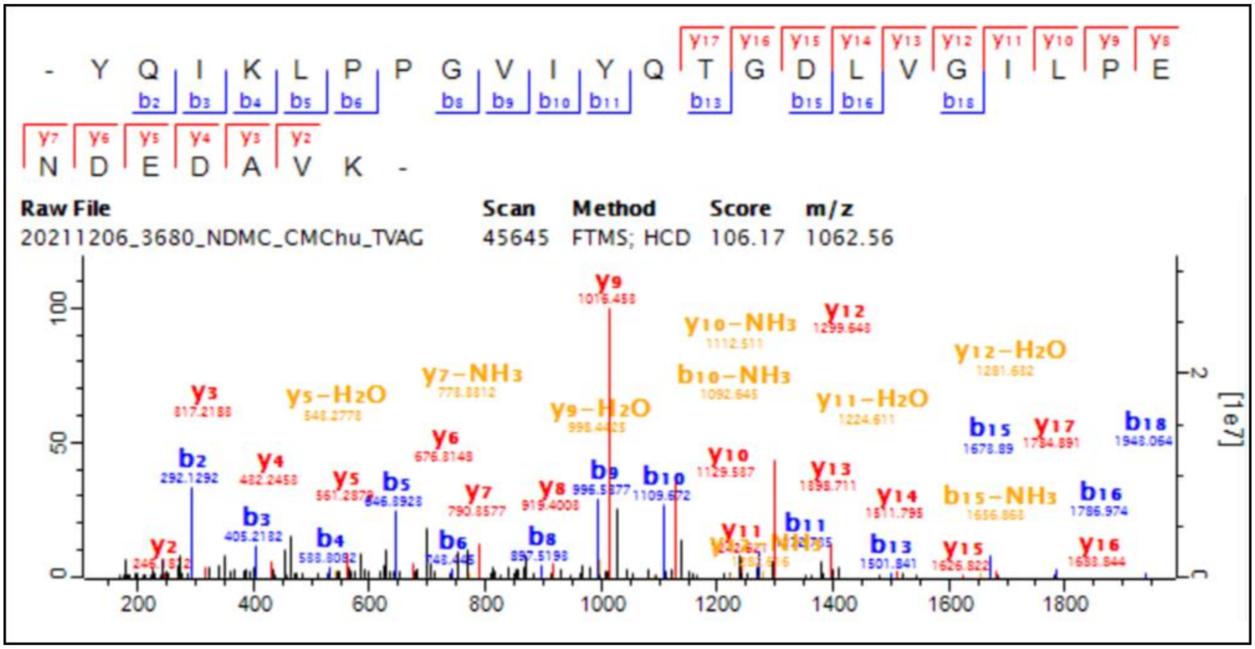

Figure S16. Mass Spectrometry protein sequence of FAD pyrophosphate binding site

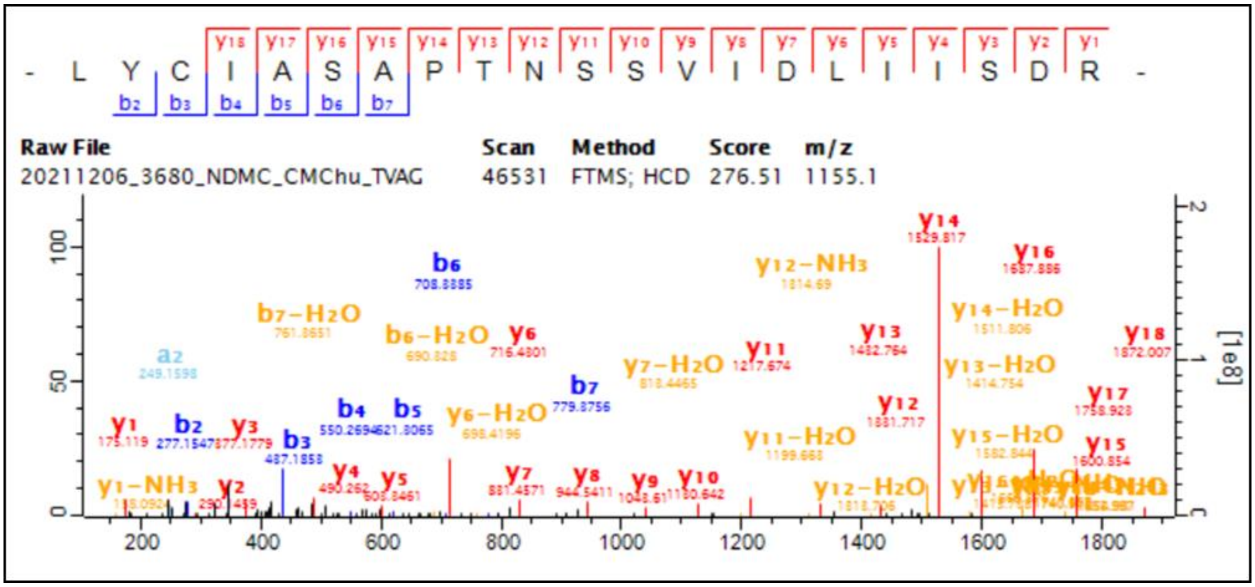

Figure S17. Mass Spectrometry protein sequence of FAD isoaaloxazine binding site

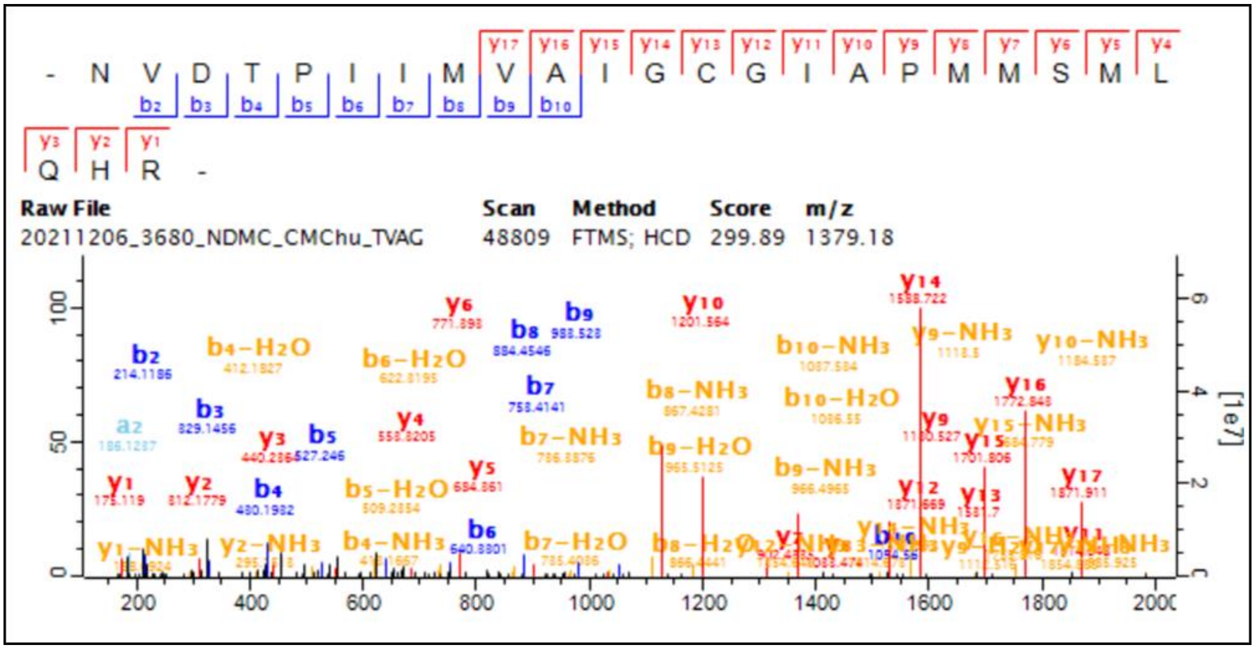

Figure S18. Mass Spectrometry protein sequence of NADPH ribose binding site

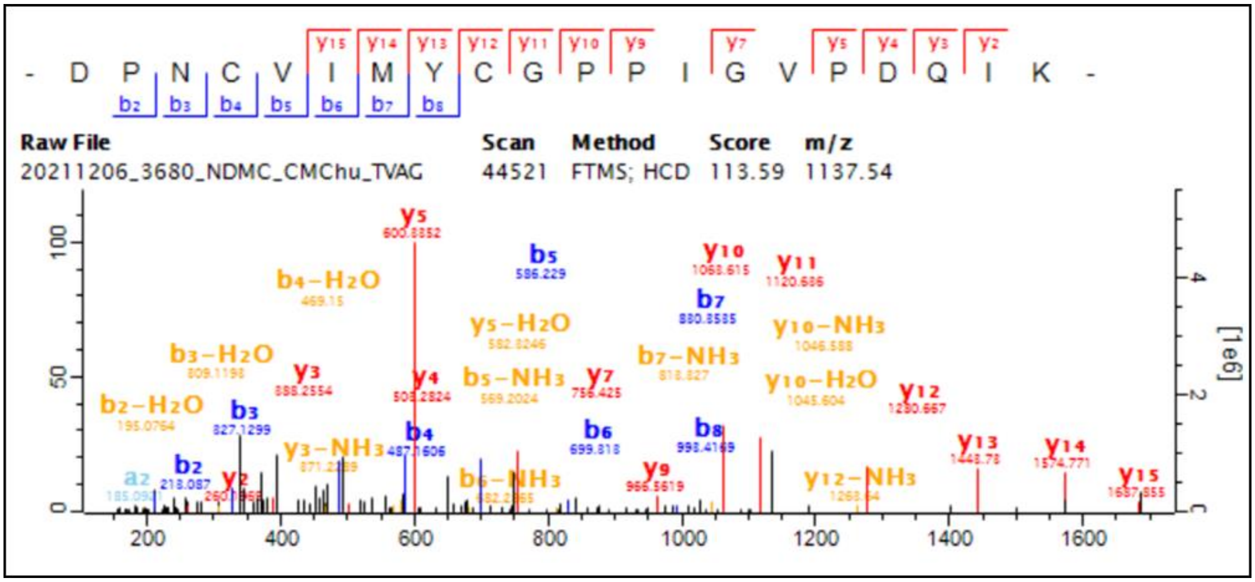

Figure S19. Mass Spectrometry protein sequence of NADPH adenine binding site

Internal validation and External validation method of our hybrid gene annotation method

Internal validation

For protein sequence X of *T. vaginalis* (with annotated function X), we assumed that *T. vaginalis* did not have function X and used Smith-Waterman alignment to compare the whole protein sequence of *T. vaginalis* with protein sequence X to verify that our bioinformatics comparison method can correctly predict function X in *T. vaginalis*. The X proteins were experimentally validated on the UniProt website, and four proteins were selected as targets for this study, with the following exclusion criteria:

1. Probable and putative protein names: these reduce the uncertainty of the validation results.
2. The number of FASTA data in the NCBI search is too small (only 2 sets of data): it does not adequately represent the cross-species protein assumed in this study.
3. Too many data items (>10,000 set of data): The comparison time is more than one month.
4. An amino acid length of less than 100: The scores below the score set in this study are included in the standard of 100 points [6,7].

External validation

For a whole protein sequence of species Y (with annotation NOS), we assumed that no NOS was found in species Y and used Smith-Waterman alignment to compare NOS sequences in other species with the sequences of species Y to verify that our bioinformatics comparison method can correctly identify NOS sequences in other species [6,7].

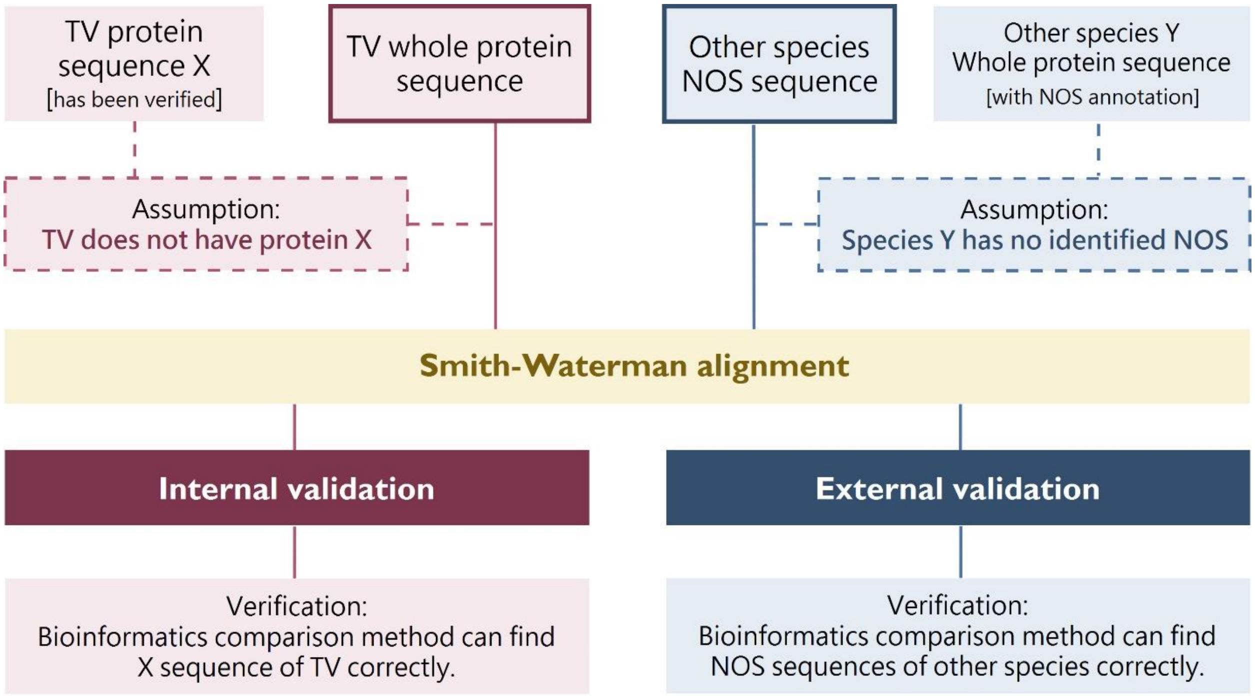

Figure S20. Internal validation and External validation of this bioinformatic method in our laboratory [7]

Table S3. The summary of the ratio of i/j and i/30 in the annotation process in our laboratory

Species	Protein	i	j	i/j	i/30	i> i	i/j>i/30	i>j and i/j>i/30	Remark
Trichomonas Vaginalis	GTF	1436	15	95.733	53.185	Y	Y	Y	internal validation
Trichomonas Vaginalis	GLK1	180	16	11.250	6.000	Y	Y	Y	internal validation
Trichomonas Vaginalis	PFP	3991	28	142.536	133.033	Y	Y	Y	internal validation
Trichomonas Vaginalis	SCL	1038	28	37.071	34.600	Y	Y	Y	internal validation
Trichomonas Vaginalis	Carbamate kinase	10249	10	1024.900	341.633	Y	Y	Y	internal validation
Trichomonas Vaginalis	**NOS**	**273**	**6**	**45.500**	**9.100**	**Y**	**Y**	**Y**	**TV NOS protein**
Trichomonas Vaginalis	insulin	8	102	0.078	0.267	N	N	N	Negative Verification
Trichomonas Vaginalis	heme oxygenase	119	33	3.606	3.967	Y	N	N	Negative Verification
Trichomonas Vaginalis	Aquaporin	3	28	0.107	0.100	N	Y	N	Negative Verification
Physarum polucephalum	NOS	5517	8	689.625	183.900	Y	Y	Y	External validation

GTF: Dolichyl-phosphate beta-glucosyltransferase; GLK1: Glucokinase 1; PFP: Pyrophosphate--fructose 6-phosphate 1-phosphotransferase; SCL: Succinate--CoA ligase

(1) i stands of the non-TV NOS protein sequences for searching candidate proteins.

(2) j stands for all TV protein sequences recognized as candidates.

After years of research in our laboratory, the rule-of-thumb of our annotation method has been formulated. We infer that the candidate sequence standard can be set by the i/j ratio (witness-to-suspect ratio). When i>j and i/j>i/30, the verification result can be established. For example, we use the human insulin sequence with TV full sequence alignment was used as a negative verification. The i/j is not greater than i/30 of human insulin to TV full sequences, so it can be inferred that Trichomonas vaginalis has no insulin protein.[1]

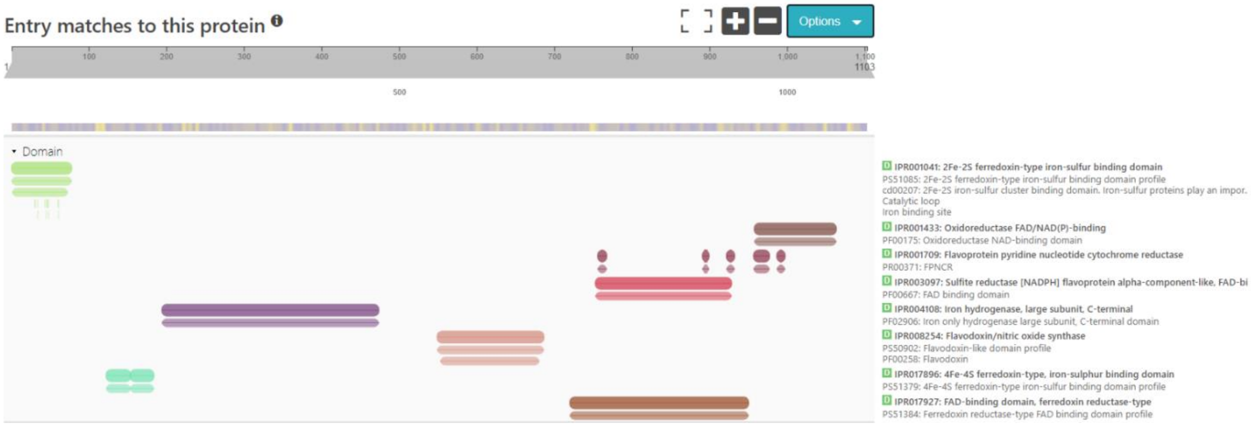

IPR001041: 2Fe-2S ferredoxin-type iron-sulfur binding domain
PS51085: 2Fe-2S ferredoxin-type iron-sulfur binding domain profile
cd00207: 2Fe-2S iron-sulfur cluster binding domain. Iron-sulfur proteins play an impor.
Catalytic loop
Iron binding site

IPR001433: Oxidoreductase FAD/NAD(P)-binding
PF00175: Oxidoreductase NAD-binding domain

IPR001709: Flavoprotein pyridine nucleotide cytochrome reductase
PR00371: FPNCR

IPR003097: Sulfite reductase [NADPH] flavoprotein alpha-component-like, FAD-bi
PF00667: FAD binding domain

IPR004108: Iron hydrogenase, large subunit, C-terminal
PF02906: Iron only hydrogenase large subunit, C-terminal domain

IPR008254: Flavodoxin/nitric oxide synthase
PS50902: Flavodoxin-like domain profile
PF00258: Flavodoxin

IPR017896: 4Fe-4S ferredoxin-type, iron-sulphur binding domain
PS51379: 4Fe-4S ferredoxin-type iron-sulfur binding domain profile

IPR017927: FAD-binding domain, ferredoxin reductase-type
PS51384: Ferredoxin reductase-type FAD binding domain profile

Figure S21. The result of protein sequences of TV NOS submitted to INTERPRO website. Domain predictions were showed.

References

1. Shevchenko, A.; Wilm, M.; Vorm, O.; Mann, M. Mass spectrometric sequencing of proteins silver-stained polyacrylamide gels. *Anal. Chem.* **1996**, *68*, 850–858; DOI:10.1021/ac950914h.
2. Gharahdaghi, F.; Weinberg, C.R.; Meagher, D.A.; Imai, B.S.; Mische, S.M. Mass spectrometric identification of proteins from silver-stained polyacrylamide gel: a method for the removal of silver ions to enhance sensitivity. *Electrophoresis* **1999**, *20*, 601–605; DOI:10.1002/(sici)1522-2683(19990301)20:3<601::aid-elps601>3.0.co;2-6.
3. Madda, R.; Chen, C.M.; Wang, J.Y.; Chen, C.F.; Chao, K.Y.; Yang, Y.M.; Wu, H.Y.; Chen, W.M.; Wu, P.K. Proteomic profiling and identification of significant markers from high-grade osteosarcoma after cryotherapy and irradiation. *Sci. Rep.* **2020**, *10*, 2105; DOI:10.1038/s41598-019-56024-7.
4. Chiang, C.H.; Wu, C.C.; Lee, L.Y.; Li, Y.C.; Liu, H.P.; Hsu, C.W.; Lu, Y.C.; Chang, J.T.; Cheng, A.J. Proteomics analysis reveals involvement of Krt17 in areca nut-induced oral carcinogenesis. *J. Proteome Res.* **2016**, *15*, 2981–2997; DOI:10.1021/acs.jproteome.6b00138.
5. Ke, C.H.; Wang, Y.S.; Chiang, H.C.; Wu, H.Y.; Liu, W.J.; Huang, C.C.; Huang, Y.C.; Lin, C.S. Xenograft cancer vaccines prepared from immunodeficient mice

increase tumor antigen diversity and host T cell efficiency against colorectal cancers. *Cancer Lett.* **2022**, *526*, 66–75; DOI:10.1016/j.canlet.2021.11.012.

6. Liu, H.L.; Chu, C.M. Genome Annotation for Nitric Oxide Synthase of *Trichomonas vaginalis* by Smith-Waterman Algorithm based on the NCBI Protein Database. M.Sc. Thesis, National Defense Medical Center, Taipei, Taiwan, 2012.
7. Yang, Y.T.; Chu, C.M. Using Smith-Waterman Alignment to Annotate Nitric Oxide Synthase in Trichomonas vaginalis. M.Sc. Thesis, National Defense Medical Center, Taipei, Taiwan, 2017.
